# Effects of age and diet on triglyceride metabolism in mice

**DOI:** 10.1016/j.jlr.2024.100706

**Published:** 2024-11-19

**Authors:** Kathryn M. Spitler, Shwetha K. Shetty, Brandon S.J. Davies

**Affiliations:** Department of Biochemistry and Molecular Biology, Fraternal Order of Eagles Diabetes Research Center, and Obesity Research and Education Initiative, University of Iowa, Iowa City, IA

**Keywords:** lipoprotein metabolism, omega-3 fatty acids, aging, triglycerides

## Abstract

Both age and diet can contribute to alterations in triglyceride metabolism and subsequent metabolic disease. In humans, plasma triglyceride levels increase with age. Diets high in saturated fats can increase triglyceride levels while diets high in omega-3 fatty acids decrease triglyceride levels. Here we asked how age and long-term diet altered triglyceride metabolism in mice. We fed male and female C57Bl/6 mice a low-fat diet, a western diet (WD), or a diet high in polyunsaturated and omega-3 fatty acids (n3D) for up to 2 years. We measured survival, body composition, plasma triglyceride levels, chylomicron clearance, and oral fat, glucose, and insulin tolerance. Triglyceride levels in mice did not increase with age, regardless of diet. Oral fat tolerance increased with age, while chylomicron clearance remained unchanged. Decreased survival was observed in WD-fed mice. Interestingly, n3D-fed mice gained more lean mass and had lower insulin levels than WD-fed or LFD-fed mice. Moreover, triglyceride uptake into the hearts of n3D-fed mice was strikingly higher than in other groups. Our data indicate that in C57Bl/6 mice, age-induced changes in triglyceride metabolism differ from those observed in humans. Mice, like humans, appeared to have decreased fat absorption with age, but in mice plasma triglyceride clearance did not decrease with age, resulting in lower plasma triglyceride levels and improved fat tolerance with age. Although a chronic diet high in omega-3 fatty acids increased insulin sensitivity and triglyceride uptake specifically into the heart, how these observations are connected is unclear.

The world population is seeing an increase in both age and obesity. In the United States, more than 20% of the population will be more than 65 years of age by the year 2030 (1). Increasing evidence has shown that changes in body weight, fat accumulation, insulin resistance, and triglyceride metabolism during aging are associated with the development of chronic metabolic diseases, such as obesity, type 2 diabetes, and hyperlipidemia (2, 3, 4). The use of animal models to model human diseases is the mainstay of scientific studies that aim to elucidate the molecular mechanisms that mediate disease. Currently, data demonstrating that mice can be used as a reliable model for obesity and metabolic disease during aging is lacking.

The consumption of high-fat food is a contributing factor to the development of metabolic diseases (5). In aging adults, the consumption of high-fat foods has increased, in part due to convenience, modern lifestyles, and the overall palatability of these foods (6). The long-term consequences of a high-fat diet can be gradual, cumulative, and intertwine with other factors such as age and obesity (7). The hallmark of obesogenic studies in mice has been the utilization of high-fat diets. However, the composition of these diets can vary across many parameters, including the source of fat, the overall percentage of fat (anywhere from 40% to 60% of kcal), and the addition of other components such as cholesterol (8). The vast majority of studies have utilized these diets for relatively short-term studies: 12 weeks to 6 months. How diets alter the metabolic phenotypes of mice over their lifetime (the average lifespan of the laboratory mouse is 2–2.5 years) is not fully understood.

In contrast to diets high in saturated fatty acids, increased consumption of omega-3 fatty acids has been proposed as a way to mitigate metabolic disease. Outcomes from the JELIS study in Japan and the Reduce-IT study, which mostly studied Caucasians, showed that high doses of the omega-3 fatty acid eicosapentaenoic acid (EPA) reduced cardiovascular risk in humans and lowered plasma triglyceride levels (9, 10), but the mechanism by which this occurs is not known. Other human studies have shown that increased consumption of omega-3 fatty acids reduces fasting VLDL, fasting plasma triglyceride levels, and postprandial triglyceride-rich lipoprotein levels (11, 12, 13). In rodent models, omega-3 fatty acids have been shown to have many potentially beneficial effects on lipid and glucose metabolism (reviewed in (14)). However, most studies in model organisms have been done in young rodents fed test diets for a limited amount of time (16 weeks or less). There is a need to examine the effects of a chronic diet in aged models.

Animal models of metabolic disease can provide valuable insight into how metabolic homeostasis can be altered by conditions such as long-term administration of a high-fat diet (15). For example, C57BL/6 mice fed a high-fat diet are classically used in the literature as models of increased fasting plasma glucose levels and insulin resistance (16). An important consideration in the utilization of animal models in biomedical research is whether they accurately model human pathologies (17). Understanding the effects of long-term high-fat diet consumption on triglyceride metabolism during the lifespan of the mouse could provide insights into the underlying mechanisms that promote metabolic disease if mice accurately reflect the human condition. However, differences between humans and mice exist and it is important to identify and address these differences. For example, in both mice and humans weight and body fat peak between mid and late life (18). In contrast, glucose levels remained relatively stable in mice until late in life when levels declined, whereas, in humans, glucose levels tend to increase throughout the course of life (18). Although several studies have observed how triglyceride metabolism is altered with age and chronic diet in humans (19, 20, 21, 22, 23, 24), there is a paucity of information regarding the utility of mice as an appropriate model of age-driven triglyceride metabolic disturbances. The aim of this study was to determine how age, in combination with a low-fat, high-saturated-fat, or high omega-3 fat diet alters triglyceride homeostasis in male and female C57BL/6 mice.

## Materials and methods

### Mice

Six-week-old C57BL/6 mice (Strain# 000664) were purchased from Jackson Laboratories. Following arrival at the University of Iowa, mice were allowed to acclimate for two weeks before initiation of study. At 8 weeks of age, mice were randomly assigned (using randomizer.org) to a low-fat diet (Teklad Diets TD.05230), a Western diet (Teklad Diets TD.200494), or a diet high in poly-unsaturated and omega-3 fatty acids (Teklad Diets TD.200495) (Table 1). 8 mice of each sex were assigned to each cohort, with the exception of the female, 2-year cohorts. For these cohorts, 10 mice were used. A total of 150 mice were used in this study. The sample size was calculated using power analysis as to have 80% power to detect 40% differences with a *P* value of 0.05 for triglyceride uptake assays. Researchers were not blinded to diet assignment. Potential confounders such as the order of measurements between diet cohorts were not controlled. Mice were initially housed 4 per cage, with the exception of the additional 2 mice/diet in the female “old” cohort which were housed 2 per cage. In some cases, animals were separated during the course of the study because of fighting or other grooming issues. All animals were maintained at 25°C with a 12:12 h light-dark cycle. Water was provided ad libitum. Mice were fed their respective diets until the time of sacrifice (age of 16 weeks, 1 year, or 2 years). Mice were weighed weekly. For food consumption studies (performed with the middle and older age cohorts only) food from each cage was weighed daily at the same time for one week. Food consumption was conducted in the middle-aged cohort at 22–28 weeks of age and in the old cohort at 41–47 weeks of age. Survival rate was tracked for mice in the two-year cohort. Death included natural death as well as humane euthanasia (100% carbon dioxide inhalation) due to severe health issues including significant weight loss, untreatable wounds or sickness, or inability to rise or ambulate. Anesthesia (when indicated below) was provided by isoflurane inhalation using a vaporizer manufactured for isoflurane. Euthanasia for terminal experiments was performed by isoflurane anesthesia followed by vital tissue harvest.

**Table 1:** Diets used in this study

	LFD	WD	n3D
**Protein, % by weight**	17.3	17.3	17.3
**Protein, % kcal from**	18.7	15.2	15.2
**Carbohydrate, % by weight**	63.5	48.5	48.9
**Carbohydrate, % kcal from**	68.7	42.7	43.0
**Sucrose, g/kg**	341	341.5	341.5
**Fat, % by weight**	5.2	21.2	21.2
**Fat, % kcal from**	12.6	42	41.9
**Saturated fat g/kg**	25.4	132.3	57.0
**Monounsaturated fat g/kg**	13.7	60.9	46.7
**Polyunsaturated fat g/kg**	9.3	8.4	73.2
**Omega-3 fat g/kg**	1.2	1.1	60.6
**omega-6:omega-3 ratio**	6.8	8	0.2

LFD, low-fat diet; n3D, omega-3-enriched diet; WD, Western Diet.

All animal procedures were approved by the Institutional Animal Care and Use Committee at the University of Iowa and were carried out in accordance with the National Institute of Health *Guide for Care and Use of Laboratory Animals*.

### Body composition analysis

Lean muscle and fat mass were determined using nuclear magnetic resonance (NMR) in mice before the start of diet (8 weeks of age), after 8 weeks of diet (16 weeks of age), and at 8 months, 12 months, 18 months, and 24 months of age. Mice were weighed and placed into a tube restraint without anesthesia. Mice were scanned with either a Bruker LF50 (mice weighing less than 50 g) or a Bruker LF90 (mice weighing over 50 g). Following NMR scanning mice were immediately returned to their home cages. NMR measurements were performed in the Fraternal Order of the Eagles Diabetes Research Center Metabolic Phenotyping Core at the University of Iowa.

### Fasting plasma triglyceride measurements

Plasma was collected in fasted (6 h) mice at the ages indicated in the figures. Blood was collected into EDTA-coated capillary tubes and plasma was collected following centrifugation (1,500 *g*, 15 min, 4°C). Plasma triglycerides were analyzed in duplicate using the Infinity Triglyceride Reagent (Thermo Scientific) as previously described (25).

### Oral fat tolerance test

Oral fat tolerance tests (FTTs) were performed in mice at 13, 49, and 75 weeks, and 2 years of age after a 12 h fast. Blood was collected into an EDTA-coated capillary tube before and 1, 2, 3, 4, and 6 h after being gavaged with olive oil (10 μl/g body weight). Plasma was collected following centrifugation (1,500 *g*, 15 min, 4°C). Plasma was stored at −80°C until all groups had been collected. Plasma triglyceride levels were measured as above using the Infinity Triglyceride Reagent (Thermo).

### Triglyceride clearance and uptake assay

^3^H-labeled chylomicrons were prepared from *Gpihbp1*^*−/−*^ mice as previously described (26). Briefly, *Gpihbp1*^*−/−*^ mice were orally gavaged with 100 μCi of [9,10-3H(N)]-Triolein (PerkinElmer, NET431001MC) suspended in olive oil. After 4 h, blood was collected via cardiac puncture and placed into a tube containing 0.5 M EDTA and placed on ice. The blood was then centrifuged at 1,500 *g* for 15 min and plasma was collected. The plasma was then centrifuged at 424,000 *g* twice for 2 h at 10°C. The chylomicrons were collected from the upper layer of the resulting supernatant, resuspended in sterile saline, and stored at 4°C. Radioactivity was measured using a PerkinElmer Liquid Scintillation Counter.

Triglyceride clearance and uptake assays were performed in mice as previously described (26). At the end of the experimental time points (16 weeks, 1 year, or 2 years of age) female and male mice were fasted (6 h), anesthetized with isoflurane, and injected retro-orbitally with ^3^H-labeled chylomicrons (100 μl). Blood was collected 1, 5, 10, and 15 min following injection and assayed for radioactivity in BioSafe II scintillation fluid (10 μl plasma/time point). Fifteen min post-injection, mice were anesthetized with isoflurane and perfused with 20 ml of PBS solution containing 0.5% Tyloxapol. Tissues (heart, liver, kidney, quadricep, gonadal white adipose tissue, subcutaneous white adipose tissue, and brown adipose tissue) were harvested and weighed. Chloroform:methanol (2:1) lipid extraction was performed on 40–90 mg of each tissue. After overnight incubation at 4°C and following phase separation with 1 ml of 2M CaCl_2_ the organic and aqueous phases were assayed for radioactivity using a Beckman-Coulter Scintillation Counter. The counts per million from each fraction were combined to give total uptake CPMs. Radiolabel CPM values were normalized to the CPM of the injected dose (measured by assaying 10% of the ^3^H-Chylomicron suspension injected into the mouse.).

### Glucose and insulin tolerance tests

Glucose tolerance tests (GTTs) were performed in fasted (6 h) mice at 14, 50, and 76 weeks and 2 years of age. Blood glucose was measured with a glucometer (OneTouch Ultra) before intraperitoneal injection of glucose (1 g/kg) and then 30-, 60-, 90-, and 120-min post-injection. At 0 and 30 min blood was also collected into EDTA-coated capillary tubes by tail vein puncture. Blood was then centrifuged at 1500 *g* for 20 min at 4°C and plasma was collected and stored at −80°C. After all blood collection, plasma insulin levels were determined using the Ultra-Sensitive Mouse Insulin ELISA Kit (Crystal Chem, 90080), according to the manufacturer’s protocols.

Insulin tolerance tests (ITTs) were performed in fasted (4 h) mice at 15, 51, and 77 weeks and 2 years of age. Blood glucose was measured with a glucometer before intraperitoneal injection of insulin (0.5U/kg, Humalin-R) and 15-, 30-, 45-, 60-, and 90-min post-injection.

### Statistics

Statistics were performed using GraphPad Prism 10. As decided a priori, we tested for outliers for each data set using the ROUT method with Q = 1%. Outliers identified in this way were removed from graphs and from statistical analysis. The total number of mice and the number of mice excluded from individual analyses are noted in supplemental Table S1. Statistical tests used for each data set are listed in the respective figure legend and in supplemental Table S1. Complete lists of *P* values are shown in supplemental Table S1.

## Results

### Experimental design

**Fig. 1:**
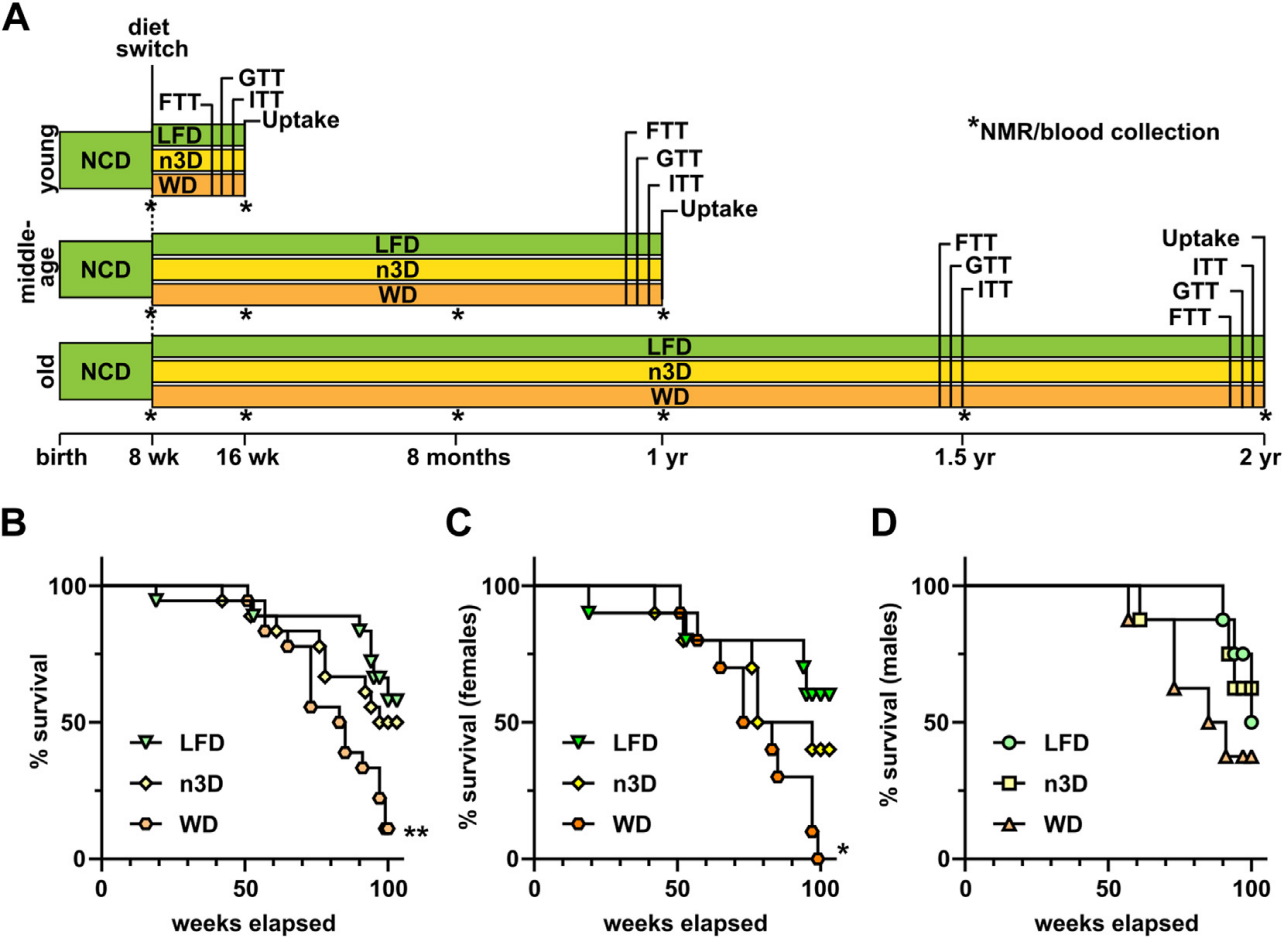
Experimental timeline and survival analysis. A: Timeline of experimental procedures for each age group. Each age group started with 8 female and 8 male mice per diet (with the exception of the female old cohort which had 10 mice/cohort). Each cohort underwent fat tolerance tests (FTT) 3 weeks prior to sacrifice, glucose tolerance tests (GTTs) 2 weeks prior to sacrifice, and insulin tolerance tests (ITTs) 1 week prior to sacrifice. Uptake assays using radiolabeled triglyceride were performed as the terminal experiment. Fasting blood was collected and body mass analysis (NMR) performed at the indicated time points (∗). B–D: Survival of combined female and male (B), female alone (C), and male alone (D) mice, in the 2-years cohort. Survival was analyzed using a log-rank (Mantel-Cox) test. ∗*P* < 0.05, ∗∗*P* < 0.01 compared to LFD mice. Complete lists of *P* values are listed in supplemental Table S1.

The experimental design for this study is shown in Fig. 1A. After 8 weeks on normal chow, mice were randomly assigned one of three diets (Table 1), a low-fat control diet (LFD; 12% kcal derived from fat), or one of two high-fat diets. The first high-fat diet was a classic “Western” diet (WD) with 42% of kcal from fat and a majority of the fat being milk fat-derived saturated fat. The second high-fat diet (omega-3 enriched high-fat diet; n3D) also had 42% of kcal from fat, but the majority of the fat was derived from fish oil and was high in polyunsaturated and omega-3 fatty acids. For each diet, mice were assigned one of three age cohorts. The young cohort was fed their respective diet for 8 weeks. The middle-aged cohort were fed their respective diets until they were 1 year old, and the old cohort until they were 2 years old. Each cohort started with 8 male and 8 female mice. Two additional mice were added to each diet of the old female cohort (giving a total of 10 mice/diet) after the early death of one mouse in this cohort led us to believe that 8 mice might not be sufficient.

### Effects of diets on lifespan in mice

In the 2-year cohort, a significant number of mice did not survive to the 2-year mark. When we plotted survival, there was no difference in survival between LFD- and n3D-fed mice (Fig. 1B). However, survival was significantly reduced in WD-fed mice compared to LFD-fed mice (Fig. 1B). When we separated mice by sex, survival was significantly decreased in females fed a WD compared to LFD (Fig. 1C). Differences in male mice did not reach statistical significance (Fig. 1D). Because of the low numbers of mice, especially WD-fed mice, that survived to the 2-years mark, we did not feel comfortable drawing conclusions from the data collected from this time point. We therefore decided to exclude the data from this time point from the main text. Moreover, in the body weight, body composition, and plasma triglyceride figures below, we included only those mice from the old cohort that survived to at least 1.5 years. However, to make the data from the complete set of mice and time points available, we have included data from all mice and all time points in the supplemental data.

### Effects of diets on body weight, food consumption, and body composition in mice

**Fig. 2:**
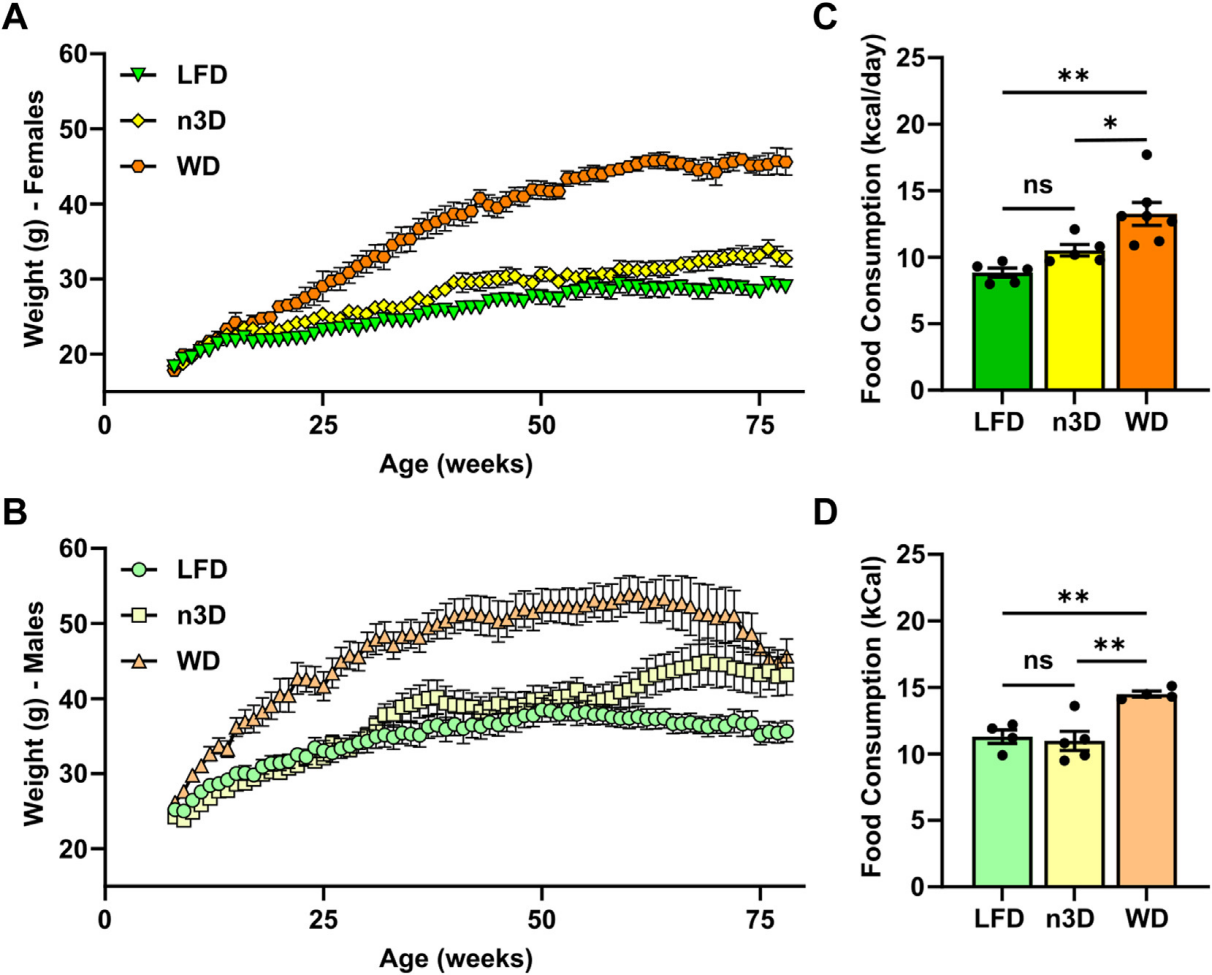
Body weight and food consumption in female and male mice. A, B: Body weights of female (A) and male (B) mice in the old cohort that survived to 1.5 years, measured weekly. C, D: Food consumption of female (C) and male (D) mice. Food consumption was measured daily for 1 week and averaged. Mice were 22–47 weeks of age when food consumption was measured. ns = not significant, ∗*P* < 0.05, ∗∗*P* < 0.01 by Tukey’s multiple comparison test after 1-way ANOVA. Complete lists of *P* values are listed in supplemental Table S1.

After the initiation of high-fat feeding, we measured body weights weekly. As expected, WD-fed males and females gained more weight than LFD fed mice (Fig. 2; supplemental Fig. S1). Body weights of LFD- and WD-fed female mice increased through 1.5 years (Fig. 2A; supplemental Fig. S1A). Not surprisingly, weights of WD-fed females dropped quickly as these mice began to die. Female n3D-fed mice had intermediate body weights. In female mice, body weights of n3D-fed mice diverged more quickly from LFD but never approached the body weights of WD-fed mice. Body weights of male mice fed either LFD or WD peaked at approximately 1 year of age and began to decline between ages 1 and 1.5 years (Fig. 2B; supplemental Fig. S1B). In males, n3D-fed mice had similar body weights to LFD-fed mice for the first 5 months on diet. Then body weights diverged, with n3D-fed mice gaining substantially more weight than LFD-fed mice. The lower weights of n3D-fed mice compared to WD-fed mice was likely due in part to lower food consumption. In both female and male mice, the caloric intake of mice fed an n3D diet was not significantly different than those fed a LFD, whereas caloric intake was higher in mice fed a WD (Fig. 2C, D).

**Fig. 3:**
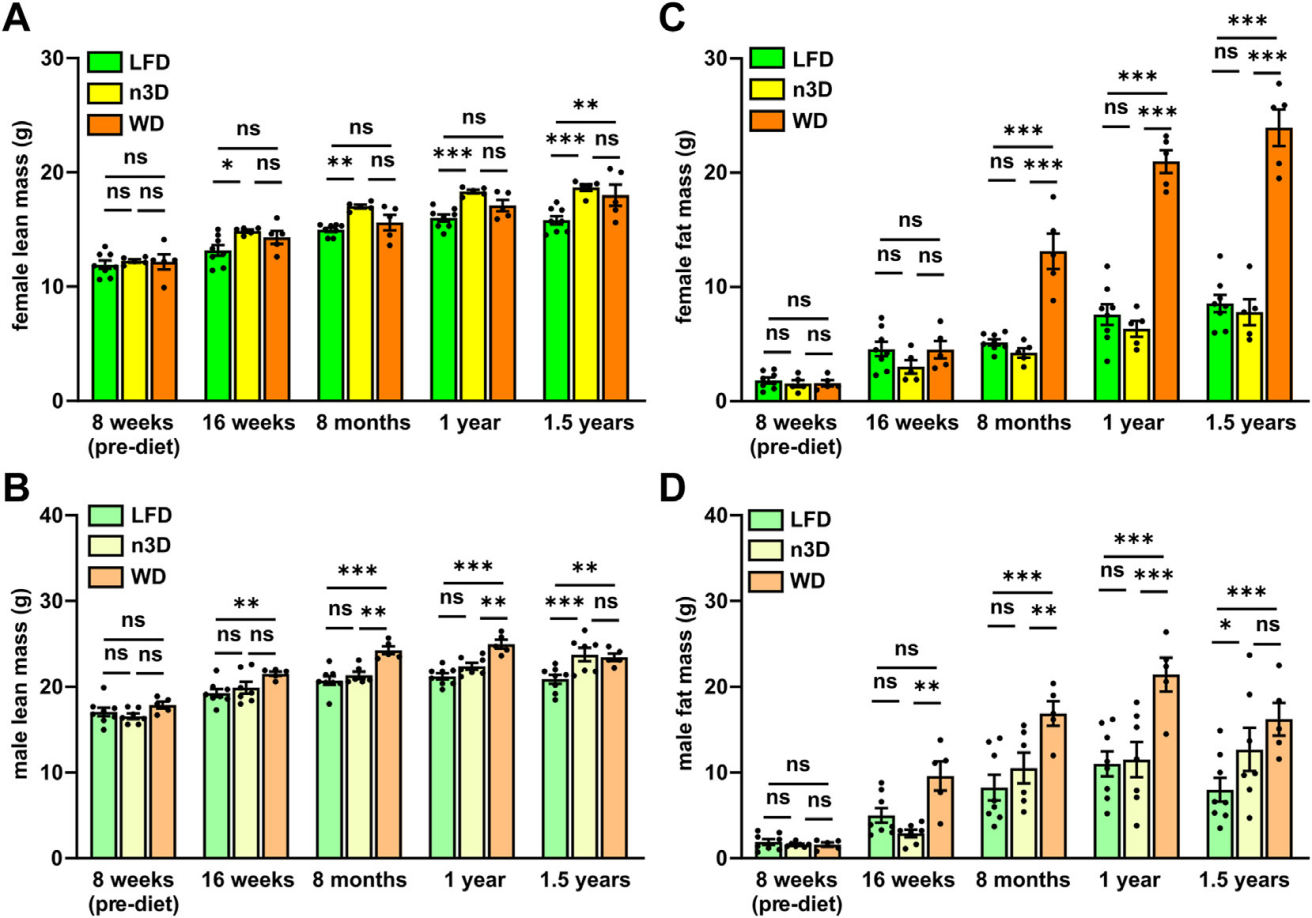
Body composition across age and diet. Lean (A, B) and fat (C, D) mass in female (A, C) and male (B, D) mice as measured by NMR in mice that survived to 1.5 years. ns = not significant, ∗*P* < 0.05, ∗∗*P* < 0.01, ∗∗∗*P* < 0.001 by Tukey’s multiple comparison test after 2-way ANOVA. Complete lists of *P* values are listed in supplemental Table S1.

In both male and female mice fed a WD, the increased body weight was reflected in both a small increase in lean mass and a large increase in fat mass as measured by NMR (Fig. 3, supplemental Fig. S2). Surprisingly, in n3D-fed mice, the increased body weight compared to LFD-fed mice appeared to be due mostly to increased lean muscle mass. In females, the fat mass of n3D-fed mice was never significantly higher than that in LFD-fed mice (Fig. 3C). In male mice, the mean fat mass of n3D-fed mice was only higher than that in LFD mice at 1.5 years of age (Fig. 3D).

### Age- and diet-induced changes in triglyceride levels

**Fig. 4:**
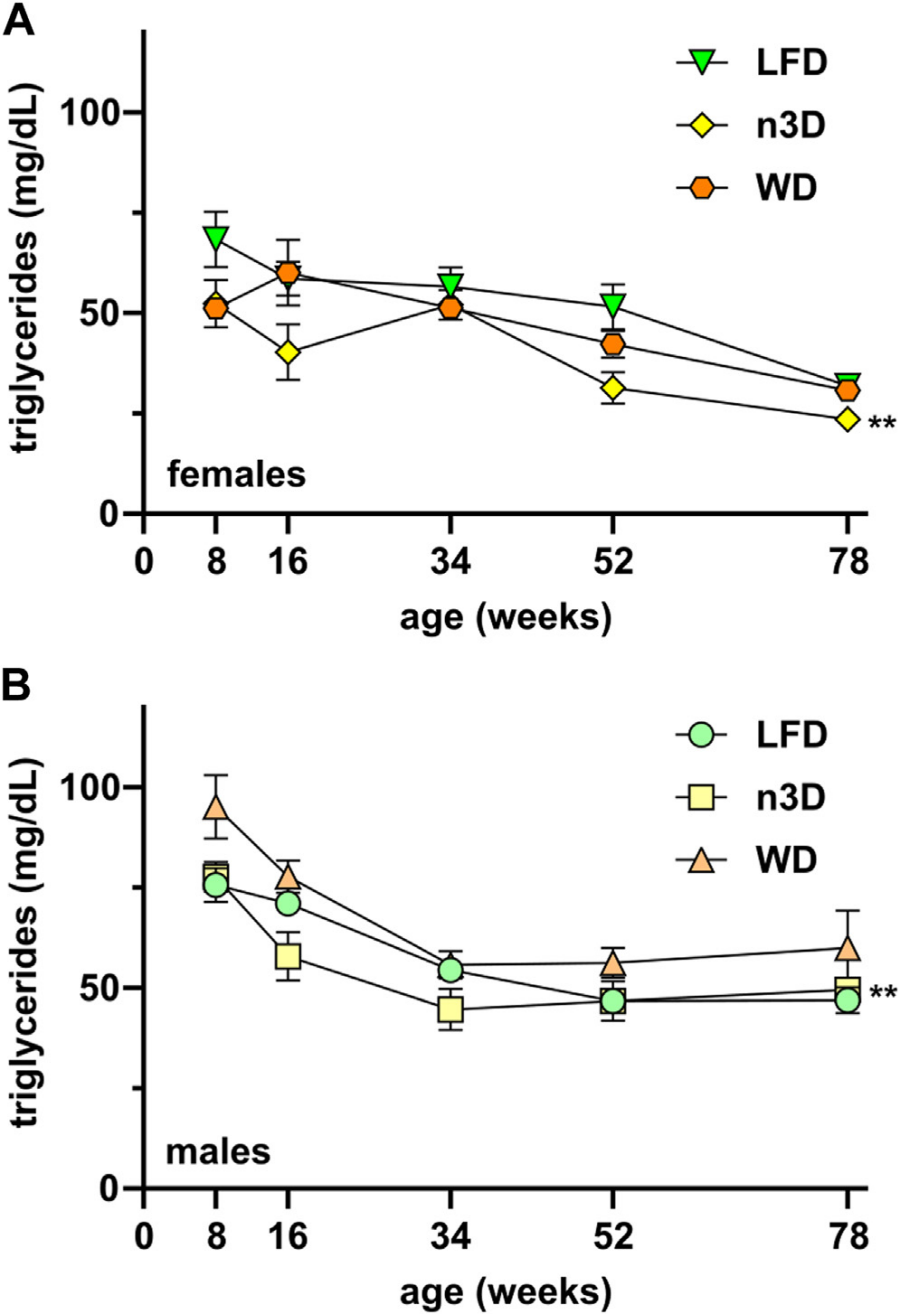
Fasting plasma triglyceride levels across age and diet. Plasma was collected from mice following a 6 h fast. Data shows plasma triglyceride levels as measured in the female (A) and male (B) mice that survived to 1.5 years. ∗∗*P* < 0.01 by mixed effects analysis. Complete lists of *P* values are listed in supplemental Table S1.

In humans, fasting triglyceride levels increase with age (19, 20, 21). To determine if plasma triglycerides in mice also change with age, we measured triglycerides in our old age cohort at 8, 16, 26, 52, 78, and 100 weeks. Contrary to our expectations, fasting plasma triglyceride levels in females on all diets decreased until 1.5 years of age (Fig. 4A). For mice that survived to 100 weeks, plasma triglyceride levels at 100 weeks were similar to those at 78 weeks (supplemental Fig. S3A). Plasma triglyceride levels in female LFD- and WD-fed mice were not different, but n3D-fed mice had modestly, but significantly lower triglycerides (Fig. 4A). Regardless of diet, in male mice fasting plasma triglyceride levels decreased until 34–52 weeks of age and then remained steady until 78 weeks (Fig. 4B). Male mice on n3D that survived to 2 years showed a rise in triglyceride levels between 1.5 and 2 years (supplemental Fig. S3B).

### Age- and diet-induced changes in oral fat tolerance

**Fig. 5:**
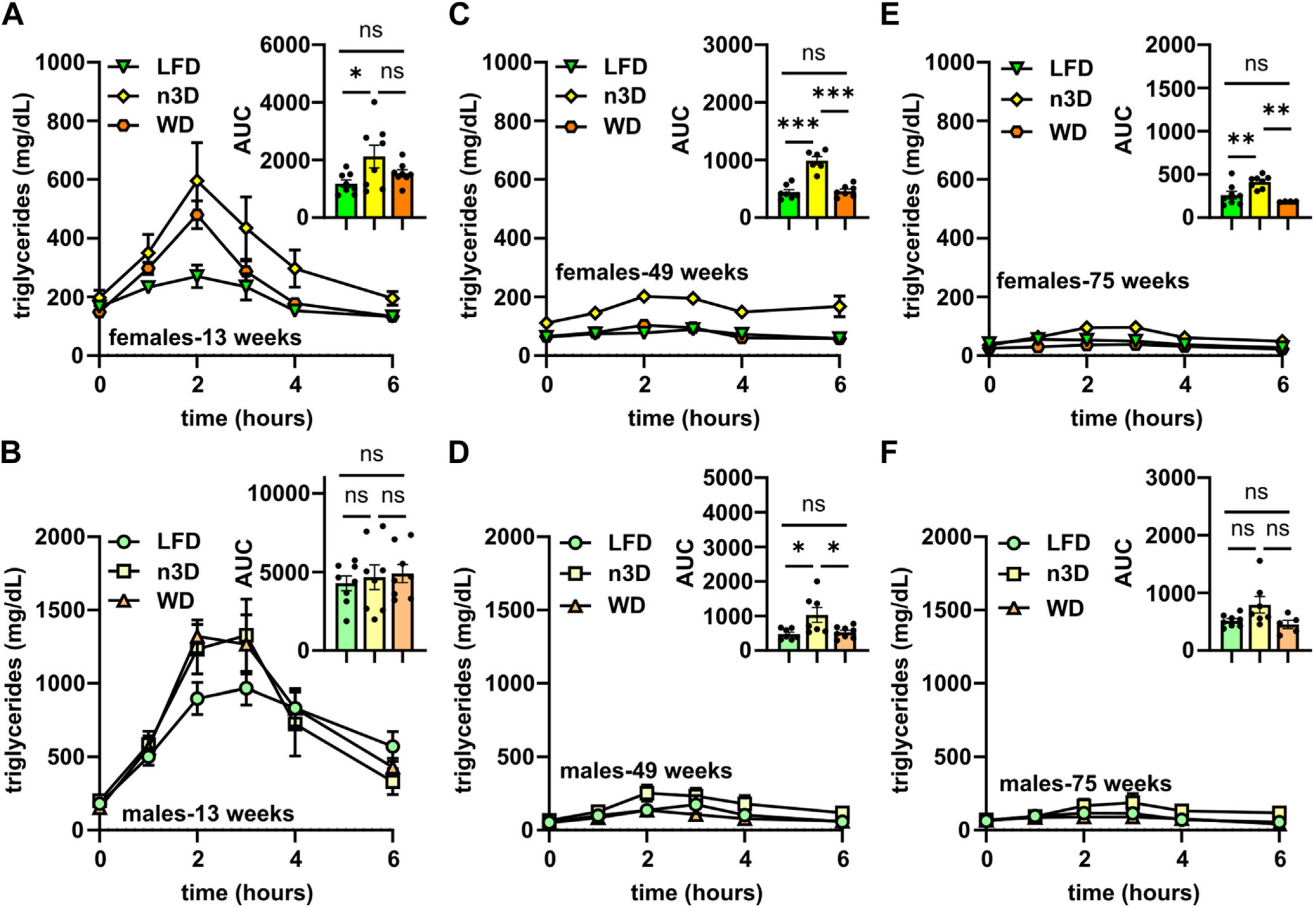
Oral fat tolerance tests. Mice were fasted (12 h) and plasma was collected before and 1, 2, 3, 4 and 6 h after olive oil gavage (10 μl/g body weight). Plasma triglyceride levels were measured at each time point in 13-week-old female (A) and male (B) mice, 49-week-old female (C) and male (D) mice, and 75-week-old female (E) and male (F) mice. Insets show area under the curve (AUC). ns = not significant, ∗*P* < 0.05, ∗∗*P* < 0.01, ∗∗∗*P* < 0.001 by Tukey’s multiple comparison test after 1-way ANOVA. Complete lists of *P* values are listed in supplemental Table S1.

Previous studies with aged humans found that clearance of serum lipid levels after a fat-loaded meal was strikingly delayed in older subjects compared with younger subjects (19, 22, 23). To elucidate if a similar delay occurs in aged mice, we performed oral fat tolerance tests (FTTs) in all 3 diet groups at 13 weeks (5 weeks on diet), 49 weeks, 75 weeks, and 2 years. In 13-week-old mice, we observed the expected peak in triglyceride levels 2–3 h after oral gavage and subsequent return of triglyceride levels towards baseline (Fig. 5A, B). In these younger mice, both the n3D and the WD-fed male and female mice had increased triglyceride levels compared to LFD-fed mice, but this difference was not statistically significant. Surprisingly, and contrary to our hypothesis, the large spike in plasma triglyceride levels following gavage was largely absent in all of the older groups (Fig. 5C–F; supplemental Fig. S4). Interestingly, in the female mice of the older cohorts, triglyceride levels after oral gavage were significantly higher in n3D-fed mice than those of LFD or WD fed mice (Fig. 5C, E).

### Effects of diets and age on triglyceride clearance and tissue triglyceride uptake

**Fig. 6:**
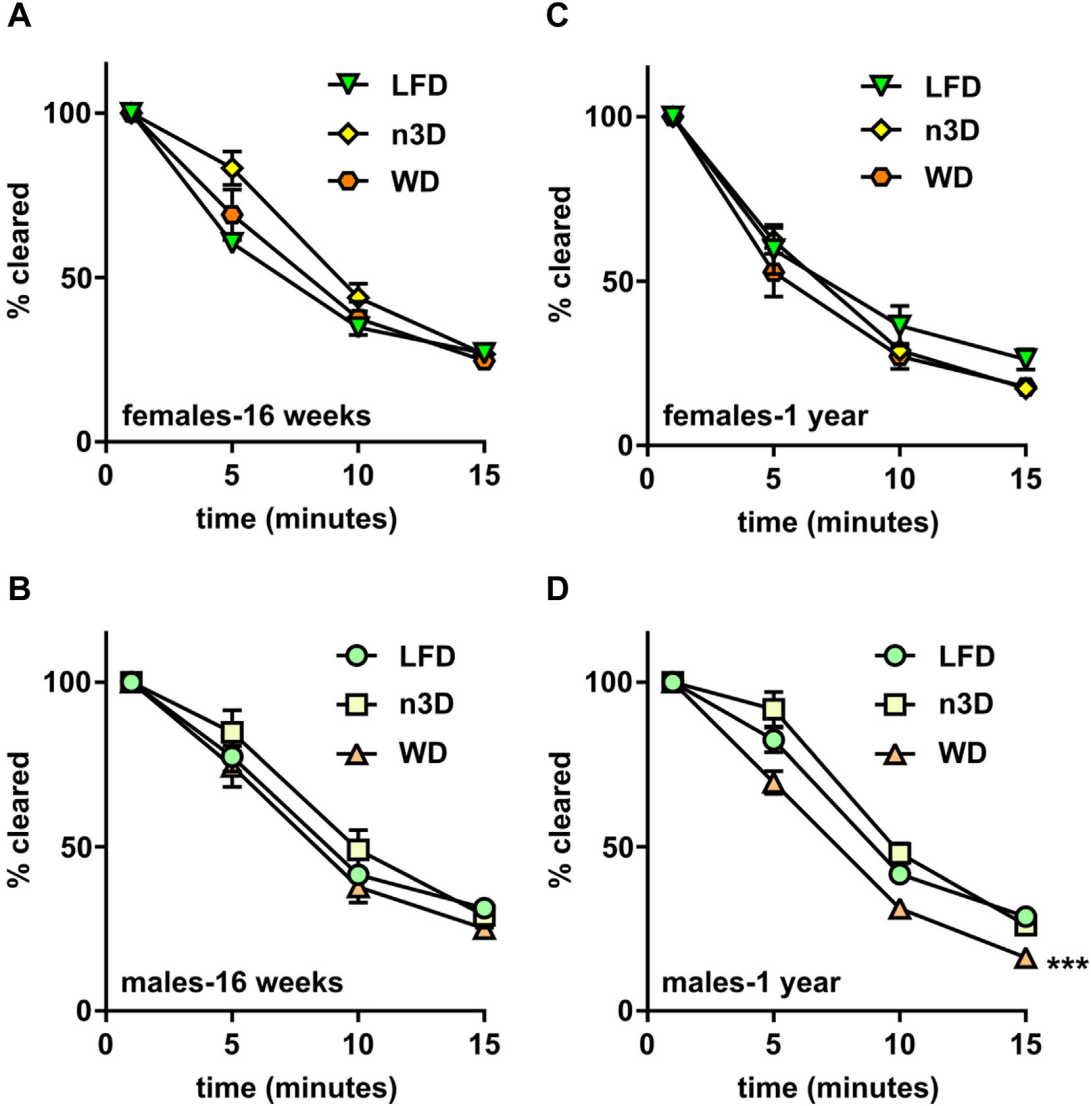
Plasma triglyceride clearance. Mice were fasted (6 h) and injected intravenously with ^3^H-triglyceride containing chylomicrons. Blood was collected after 1, 5, 10, and 15 min after injection to measure clearance of radiolabel from the plasma. Points represent radiolabel remaining in the plasma as a percentage of the 1-min time point (means ± SEM) in 16-week-old female (A), 16-week-old male (B), 1-year-old female (C), and 1 year old male (D) mice. ∗∗∗*P* < 0.001 for diet by repeated measures 2-way ANOVA. Complete lists of *P* values are listed in supplemental Table S1.

The partitioning of triglycerides between different adipose tissue depots and other peripheral locations differs not only between sexes but also during aging (27, 28, 29, 30, 31, 32). To better understand how sex, age, and diet can lead to alterations in triglyceride storage and partitioning we performed plasma triglyceride clearance and tissue uptake assays. Mice were injected with chylomicrons containing radiolabeled triglyceride, and the clearance of radiolabel from the bloodstream was measured over 15 min. After 15 min, mice were treated with Tyloxapol to block further lipolysis of lipoproteins, and tissues were collected and assessed for radiolabel uptake. The profile of triglyceride clearance from the blood was similar across all ages (Fig. 6; supplemental Fig S5). There were no diet-induced differences in plasma triglyceride clearance in female mice (Fig. 6A, C). In male mice, there were no diet-induced differences observed in the young cohort (Fig. 6B), but in the middle-aged male cohort, WD-fed mice cleared triglycerides faster than LFD- or n3D-fed mice (Fig. 6D).

**Fig. 7:**
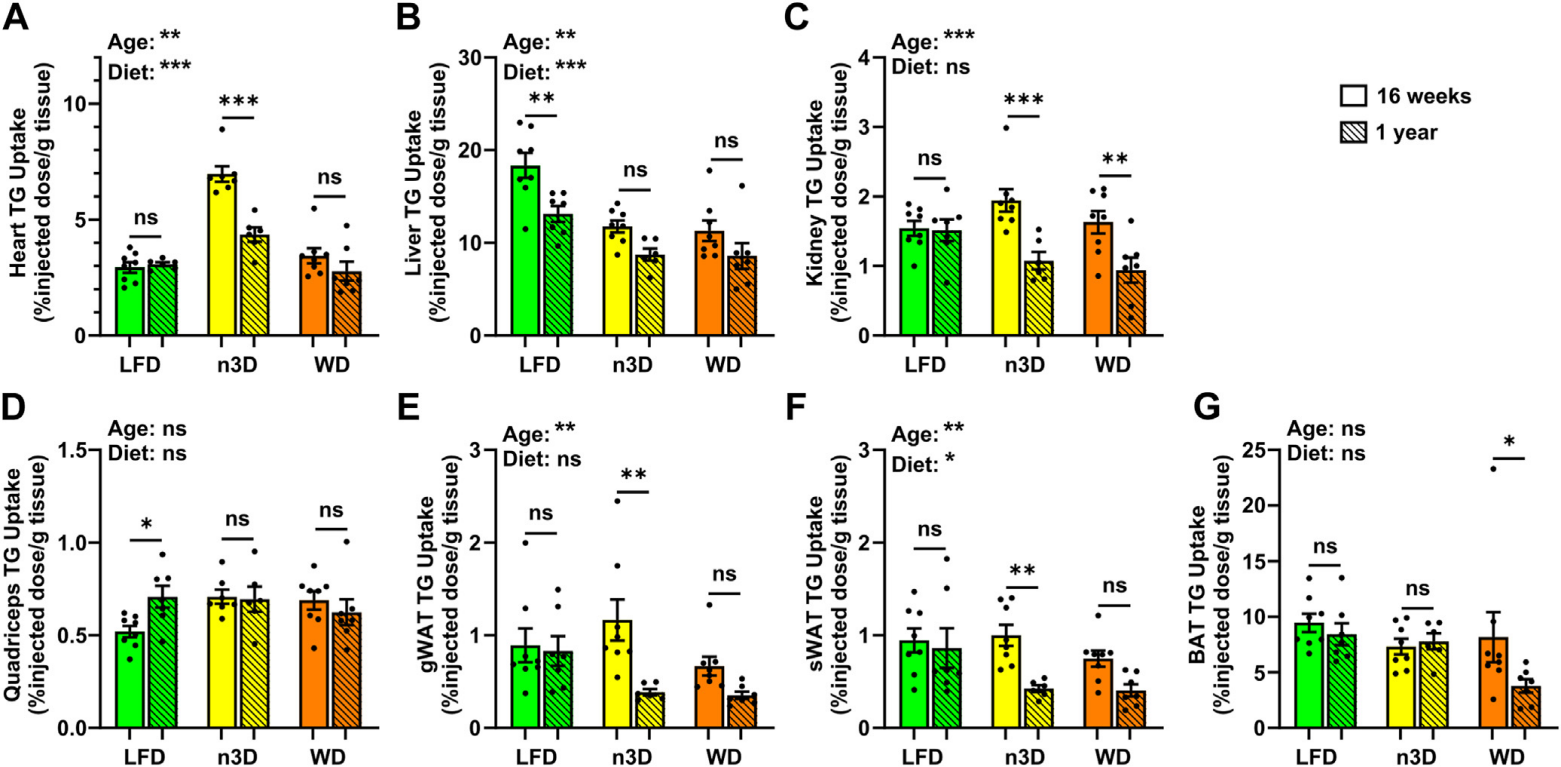
Tissue triglyceride uptake in female mice. At 16 weeks or 1 year of age female mice were fasted (6 h) and injected intravenously with ^3^H-triglyceride containing chylomicrons. After 15 min, tissues were harvested and uptake of radiolabel (% injected dose/g tissue) was measured in heart (A), liver (B), kidney (C), quadriceps muscle (D), gonadal white adipose tissue (gWAT) (E), subcutaneous white adipose tissue (sWAT) (F), and brown adipose tissue (BAT) (G). Bars represent mean ± SEM. ns = not significant, ∗*P* < 0.05, ∗∗*P* < 0.01, ∗∗∗*P* < 0.001 by 2-way ANOVA and subsequent Tukey’s multiple comparison test. Complete lists of *P* values are listed in supplemental Table S1.

**Fig. 8:**
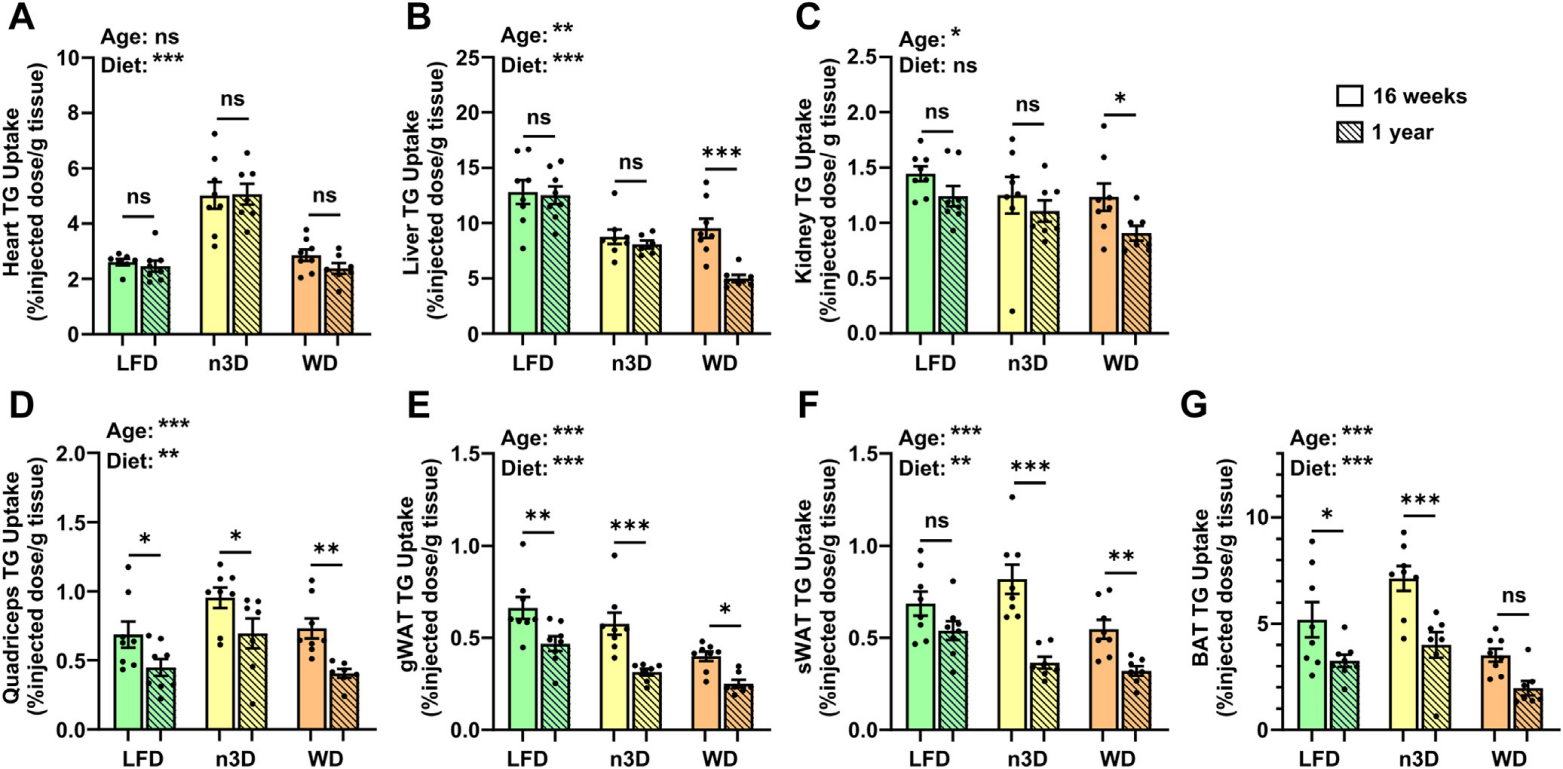
Tissue triglyceride uptake in male mice. At 16 weeks or 1 year of age mice were fasted (6 h) and injected intravenously with ^3^H-triglyceride containing chylomicrons. After 15 min, tissues were harvested and uptake of radiolabel (% injected dose/g tissue) was measured in heart (A), liver (B), kidney (C), quadriceps muscle (D), gonadal white adipose tissue (gWAT) (E), subcutaneous white adipose tissue (sWAT) (F), and brown adipose tissue (BAT) (G). Bars represent mean ± SEM. ns = not significant, ∗*P* < 0.05, ∗∗*P* < 0.01, ∗∗∗*P* < 0.001 by 2-way ANOVA and subsequent Tukey’s multiple comparison test. Complete lists of *P* values are listed in supplemental Table S1.

Except for the quadriceps in LFD-fed females, on a per gram tissue basis, triglyceride uptake either stayed the same or decreased from the young-age cohort to the middle-age cohort in all tissues and on all diets (Figs. 7 and 8). The most significant age-mediated decreases in triglyceride uptake were seen in white adipose tissue, particularly those fed high-fat diets (Figs. 7E, F and 8E, F). It is important to note that even though uptake went down on a per gram basis in many tissues, because of the increased size of these tissues with age, the total amount of triglycerides entering these tissues either stayed the same or increased (supplemental Fig. S6). In the liver, reduced triglyceride uptake (on a per gram tissue basis) was observed for both high-fat diets compared to the LFD (Figs. 7B and 8B); however, these differences were less striking when calculating uptake on a whole tissue basis (supplemental Fig. S6). The most striking effects of diet on triglyceride uptake were found in the heart. In both male and female hearts, n3D-fed mice took up substantially more radiolabel than either LFD- or WD-fed mice (Figs. 7A and 8A). This was true across age groups and was true whether calculating uptake on a per gram of tissue (Figs. 7A and 8A) or a total tissue (supplemental Fig. S6) basis. When analyzing the surviving old cohort mice, triglyceride uptake did go up in some tissues, but again the low numbers, questionable health status, and lack of surviving WD-fed female mice in these cohorts make it difficult to interpret the data (supplemental Fig. S7 and S8).

### Effects of diets and age on glucose metabolism

**Fig. 9:**
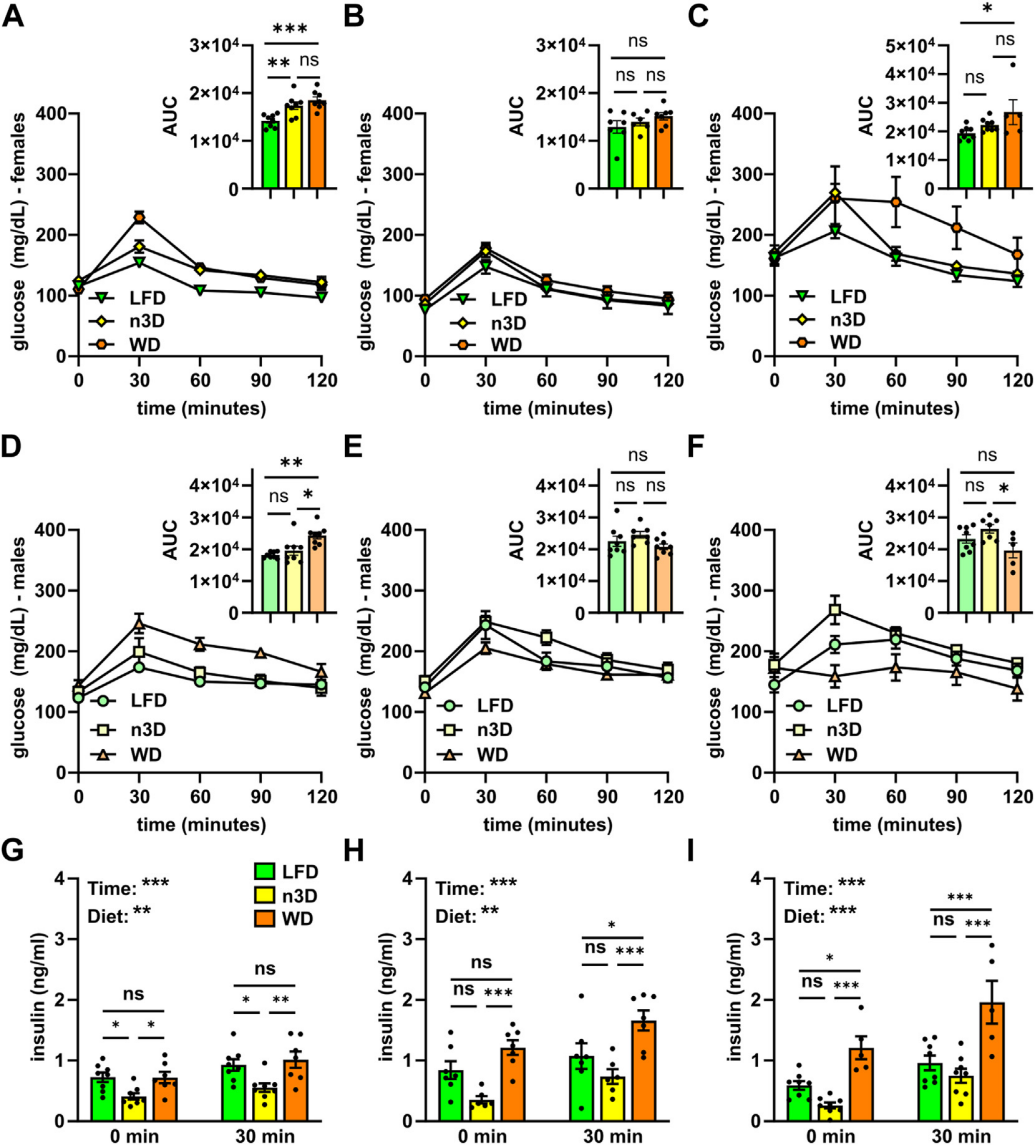
Glucose tolerance tests and plasma insulin levels. A–F: Glucose tolerance tests were performed on fasted (6 h) female (A–C) and male (D–F) mice at 14 (A, D), 50 (B, E), and 76 (C, F) weeks of age. Mice were injected with glucose (1 g/kg) and blood glucose concentrations were measured over 2 h. Points represent glucose levels (means ± SEM) at each respective time point. Insets show area under the curve (AUC). ns = not significant, ∗*P* < 0.05, ∗∗*P* < 0.01, ∗∗∗*P* < 0.001 by Tukey’s multiple comparison test after 1-way ANOVA. G–I: Plasma insulin levels before and 30 min after glucose injection in 14- (G), 50- (H), and 76- (I) week-old female mice. Bars represent mean ± SEM. ns = not significant, ∗*P* < 0.05, ∗∗*P* < 0.01, ∗∗∗*P* < 0.001 by 2-way ANOVA and subsequent Tukey’s multiple comparison test. Complete lists of *P* values are listed in supplemental Table S1.

To understand how diet and age affect glucose homeostasis in mice, we performed glucose tolerance tests (GTTs) and insulin tolerance tests (ITTs) at 14–15 weeks (young cohort, 6–7 weeks on diet), 50–51 weeks (middle-age cohort), 76–77 weeks (old cohort), and 2 years of age (old cohort) in both males and females. After 6 weeks on WD, there was a significant loss of glucose tolerance compared to LFD in both male and female mice (Fig. 9A, D). Female n3D-fed mice had reduced glucose tolerance, but male n3D-fed mice were not significantly different from LFD-fed mice (Fig. 9A). Surprisingly, after 1 year, there were no statistical differences in glucose tolerance across diets (Fig. 9B, E), though it should be noted that by 1.5 years there was substantial variability in the WD-fed groups (Fig. 9C, F).

**Fig. 10:**
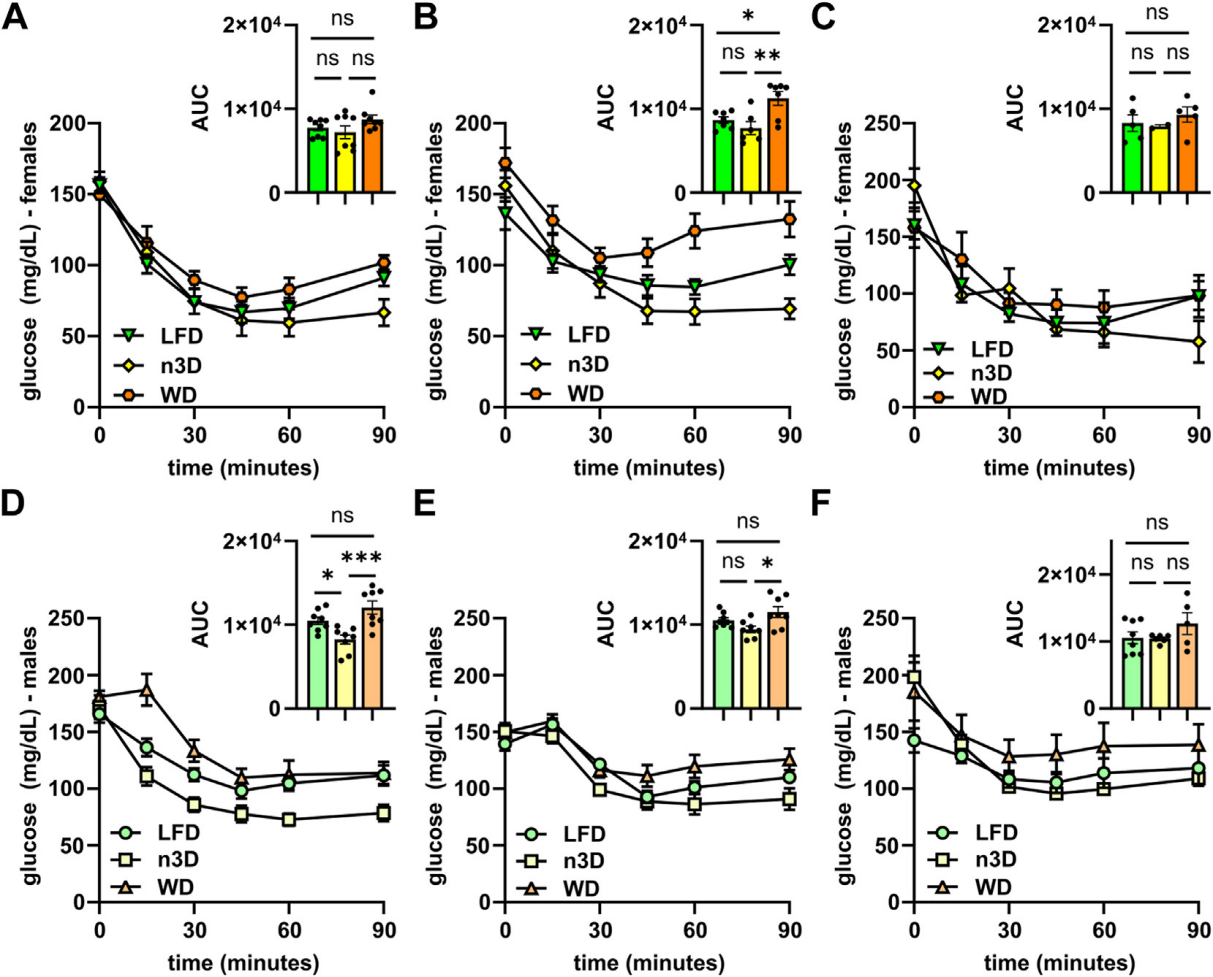
Insulin tolerance tests. Insulin tolerance tests were performed in fasted (4 h) female (A–C) and male (D–F) mice at 15 (A, D), 51 (B, E), or 77 (C, F) weeks of age. Mice were injected with 0.5 U/ml of human insulin (Humalin-R) and blood glucose concentrations were measured over 90 min. Points represent glucose levels (means ± SEM) at each respective time point. Insets show area under the curve (AUC). ns = not significant, ∗*P* < 0.05, ∗∗*P* < 0.01, ∗∗∗*P* < 0.001 by Tukey’s multiple comparison test after 1-way ANOVA. Complete lists of *P* values are listed in supplemental Table S1.

As part of our GTT assays, we measured insulin levels in female mice before and 30 min after glucose injection. Interestingly, n3D-fed mice had lower insulin levels both prior to and 30 min after glucose injection, especially compared to WD-fed mice (Fig. 9G–I). This was true at all ages (Fig. 9G–I, supplemental Fig S9), though mouse numbers were low at 2 years. The reduced insulin levels in n3D-fed mice, despite similar glucose tolerance, suggested that these mice might have increased insulin tolerance. Indeed, insulin tolerance tests suggested that n3D-fed mice were more insulin sensitive than mice fed the other two diets (Fig. 10; supplemental Fig S10). Increased insulin sensitivity in n3D-fed mice was also supported by the fact that when performing ITTs on 1.5-year-old female mice, only 2 of the 8 n3D-fed mice were able to finish the time course as all others had to be given a bolus of glucose due to hypoglycemia. 3 of 8 LFD-fed mice also had to be given glucose.

## Discussion

In this study, we fed mice three different diets for up to two years to examine how age and diet affected triglyceride metabolism in mice. Our major findings include 1) a high-fat western diet reduces life span; 2) whereas in humans plasma triglyceride levels increase with age and triglyceride clearance after a fatty meal decreases, the opposite appears to be true in C57Bl/6 mice where both fasting triglyceride levels and postprandial spikes in plasma triglyceride levels were lower in older mice than those in young mice; and 3) a diet high in polyunsaturated and omega-3 fat increased the uptake of triglyceride-derived fatty acid into the heart and led to an increase in lean muscle mass.

A Western diet is known to exacerbate several metabolic diseases in humans and is thought to enhance the risk of mortality (33, 34, 35). In mice, a Western diet has been shown to increase mortality in combination with pathologies such as sepsis (36), genetically induced hepatic steatosis (37), and taurocholate-induced necrotizing pancreatitis (38). Here we show that a Western diet without any other interventions decreased survival in mice when compared to a low-fat diet. Interestingly this change in survival was more pronounced in female mice than in male mice.

A major goal of this study was to understand how triglyceride metabolism changes with age in mice and compare these changes to those previously observed in humans. In humans, both fasting and postprandial triglyceride levels are greater in men than in women, and in both sexes, triglyceride levels increase with age (20). Although we did find that fasting and postprandial triglyceride levels were higher in male mice than female mice, mostly notably at younger ages, unlike humans, plasma triglyceride levels did not increase with age. Instead, we found that for both male and female mice, fasting triglyceride levels were lower in old mice versus young mice. This was consistent with the findings of Houtkooper *et al.* who found increased free fatty acids and decreased triglyceride levels in aged (22 months) mice compared to young (3 months) mice (39), but differed from the findings of Xiong *et al.* who found that plasma triglyceride levels were greater in 20 month-old mice than 2-month-old mice (40). Interestingly, we also found that in our mice, triglyceride levels decreased with age regardless of diet, and mice fed a Western diet did not have significantly higher triglyceride levels than those fed a low-fat diet.

As with fasting triglyceride levels, postprandial triglyceride phenotypes also differed between our mouse cohort and what has been observed in humans. Several human studies have shown that the post-prandial increase in triglyceride levels after a high-fat meal is both higher and longer in aged individuals than in young individuals (19, 22, 23). Here we found that older male and female mice had a much lower rise in plasma triglycerides after a high fat gavage. This lower triglyceride rise could be mediated by lower fat absorption and subsequent secretion of chylomicrons into the circulation, or it could be mediated by faster triglyceride clearance. Yamamoto *et al.* also found that the postprandial increase in plasma triglycerides was lower in aged mice and concluded that absorption was lower based on finding lower levels of pancreatic lipase activity (41). It should be noted that in that study, plasma triglyceride clearance was not measured, postprandial triglyceride levels were only measured at the 3 h mark, and experiments were done only in male mice (41). Despite the limitations of this previous study, our data also suggest a decrease in fat absorption in both male and female aged mice, as our triglyceride clearance assays did not show a significant difference in triglyceride clearance rate with age (see Fig. 6). Interestingly, humans also seem to have decreased fat absorption rates with age (42, 43), suggesting that the increase in postprandial triglyceride levels in humans is likely due to dysfunctional clearance. Indeed, Vinagre *et al.* found that LPL activity in post-heparin plasma was reduced in aged individuals and that after injection of chylomicron-like emulsions, clearance of remnant particles was reduced (23). Overall, our data suggest that mice may have a similar decrease in fat absorption when compared to humans, but that mice maintain functional triglyceride clearance with age whereas humans do not. As a result, post-prandial triglyceride levels increase with age in humans but decrease with age in mice.

That aging mice differ from aging humans in respect to triglyceride metabolism is perhaps not that surprising given the known differences between mice and human lipoprotein homeostasis. For example, mice, unlike humans, have far more HDL than LDL (44) and lack the cholesterol-ester transport protein found in humans (45). The low levels of LDL in mice suggest that compared to humans, mice clear VLDL remnants more efficiently, converting less VLDL to LDL. Just as genetic manipulation has allowed mouse models that more closely reflect human LDL and plasma cholesterol levels, it may be possible that similar mouse models might more closely resemble the triglyceride phenotypes found in aging humans.

Some of the most intriguing findings of this study come from the cohort of mice fed a diet high in polyunsaturated and omega-3 fatty acids. Chronic low-grade inflammation is associated with aging (46) and obesity (47). This heightened inflammatory state presents a potential therapeutic target for disturbances in glucose and lipid metabolism during aging. Omega-3 polyunsaturated fatty acids have anti-inflammatory properties and might prove to be a therapeutic agent (48). A meta-analysis found omega-3 treatment reduced C-reactive protein and IL-6 plasma levels in adults 45 or older (49). Low consumption of seafood-derived omega-3 fats is associated with an increased risk of diet-related cardiometabolic death (50), while higher circulating levels of omega-3 fatty acids are associated with lower total mortality and lower risk of cardiovascular deaths in older adults (51). Omega-3 fats have become increasingly studied for their role in metabolic disease; however, few studies have examined long-term treatment with Omega-3 fatty acids and metabolic health in mice. We found that n3D-fed mice had lower body weights than WD-fed mice, and unlike WD-fed mice did not have significantly reduced survival compared to LFD-fed mice. Increased lean mass was observed in n3D-fed mice, particularly female mice, compared to LFD-fed mice but not increased fat mass compared to LFD-fed mice. These mice also appeared to be more insulin sensitive and have lower insulin levels than LFD- or WD-fed mice. Moreover, both male and female n3D-fed mice had a striking increase in triglyceride uptake into the heart. The low body weights in n3D-fed mice are likely a result of the lower calorie consumption observed in these mice compared to WD-fed mice. Likewise, the improved survival, lower insulin levels, and improved insulin sensitivity could be ascribed to the lower calorie consumption and the anti-inflammatory effects of omega-3 fatty acids. However, it is mechanistically unclear how an omega-3 fatty acid diet leads to increased fatty acid uptake into the heart and an increase in lean mass rather than fat mass. It is also unclear if these two phenotypes are related to each other or to any of the other metabolic phenotypes observed in these mice. It is possible that the increased uptake into the heart drove increased lean muscle mass. After 1 year on diet, the hearts of female n3D-fed mice were significantly larger than those of LFD-fed mice (170.8 vs. 137.1 mg; *P* = 0.004). A difference in heart weights between n3D-fed and LFD-fed mice was not observed in males.

Our study has several limitations. Although we performed assays on 2-year-old mice, the number of mice that died before reaching two years of age left the assays performed at this time point underpowered. Hence our decision to move all data from this time point to supplemental data, and we are unable to draw any conclusions about triglyceride metabolism at the oldest time point. Moreover, although we found that WD-fed mice had reduced survival, we did not investigate the causes of death in these mice. Another limitation is that we examined only three diets. Although we carefully chose the diets used in this study, there are, of course, several different types of high-fat diets utilized in studies of diet-induced obesity and insulin resistance. It is possible that certain diets could have very different effects on triglyceride metabolism. Experimentally, it is important to note that by necessity, experiments with the different sets of mice were performed at different times. While for some experiments (e.g., plasma triglycerides, insulin, etc...) samples were frozen and then all samples assayed at the same time, this was not possible for experiments such as triglyceride clearance assays where preparation of radiolabeled chylomicrons is performed freshly for each experiment. While we attempted to keep experimental conditions as consistent as possible, variations due to batch effects are possible. An additional limitation is that, while oral gavage is widely used as a means to deliver drugs and therapeutics to mice, there can be negative side effects. In this study, the testing of oral fat tolerance was performed by gavaging mice with 10 μl olive oil/g body weight. As the mice gained weight the amount of each olive oil gavage also increased. These larger doses could potentially lead to gastrointestinal issues that limit triglyceride absorption and could have affected our findings at later time points. Finally, there are a number of metabolic parameters that we did not measure. For example, although our data suggest that fat absorption decreases with age, we did not directly measure fat absorption. A more complete measurement of different metabolic parameters as well as gene and protein expression could provide a more comprehensive picture of age- and diet-induced changes in triglyceride metabolism.

## Data availability

The data that support the findings of this study are available from the corresponding author upon reasonable request.

## Supplemental data

This article contains supplemental data.

## Conflict of interest

The author is an Editorial Board Member/Editor-in-Chief/Associate Editor/Guest Editor for *Journal of Lipid Research* and was not involved in the editorial review or the decision to publish this article.
