# Supplementary material for: Effects of age and diet on triglyceride metabolism in mice
Source: J Lipid Res. 2024 Nov 19;66(1):100706. doi: 10.1016/j.jlr.2024.100706 (PMC11730548; doi:10.1016/j.jlr.2024.100706)
Supplement: supplemental Figs. S1–S10 and Table S1 [file mmc1.pdf]

SUPPLEMENTARY DATA

Supplementary Figure 1

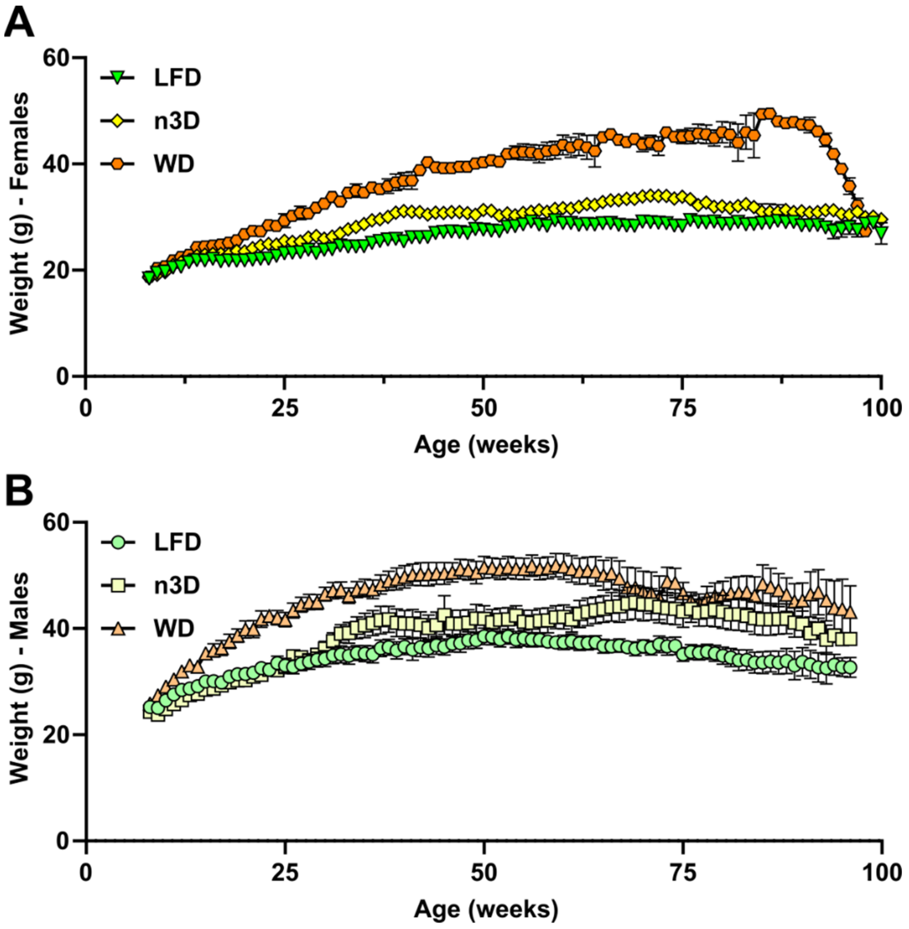

Supplementary Figure 1: Body weight of all female and male mice in the old cohort. Body weights of female **(A)** and male **(B)** mice in the 2-year cohort, measured weekly.

Supplementary Figure 2

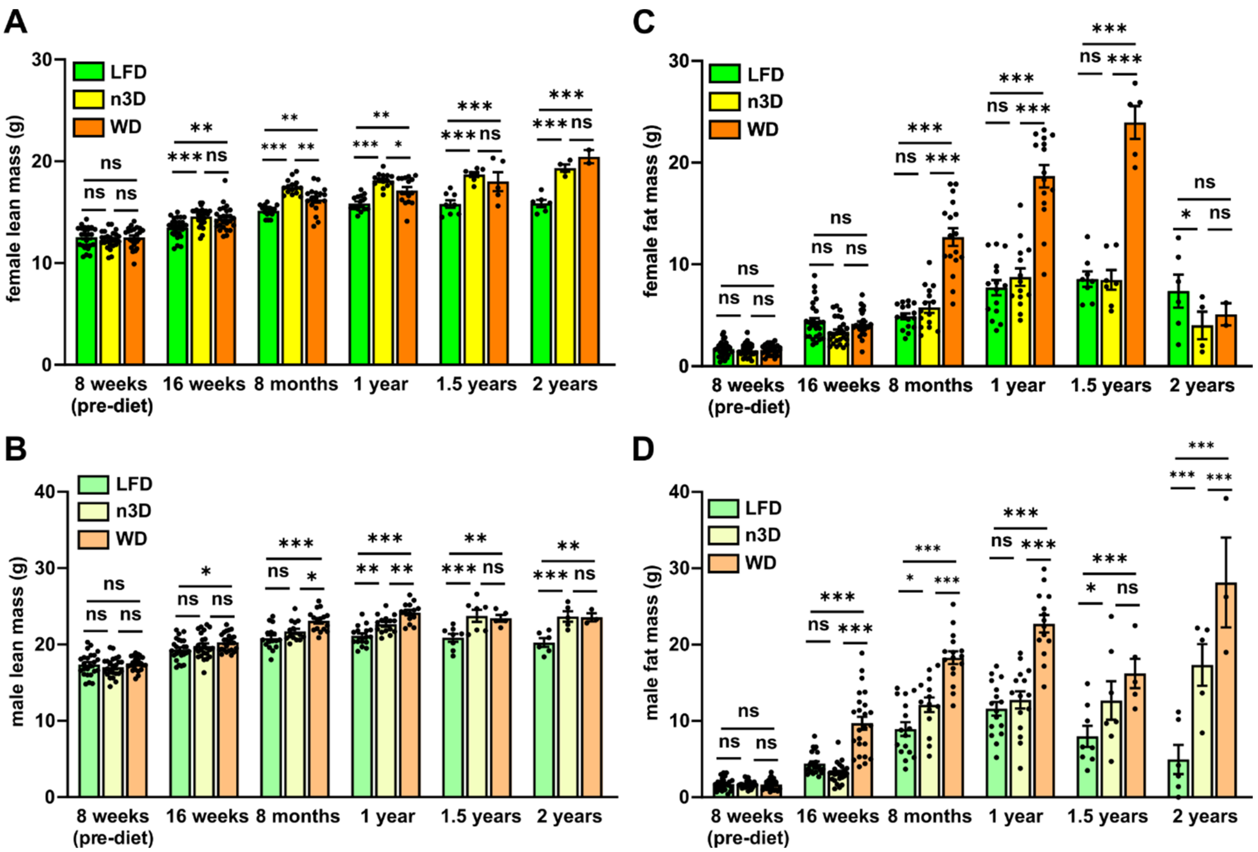

Supplementary Figure 2: Body composition across age and diet. Lean (A-B) and fat (C-D) mass in all female (A,C) and male (B,D) mice as measured by NMR. ns=not significant, *p<0.05, **p<0.01, ***p<0.001 by Tukey's multiple comparison test after 2-way ANOVA. Complete lists of p values are listed in Supplementary Table 1.

Supplementary Figure 3

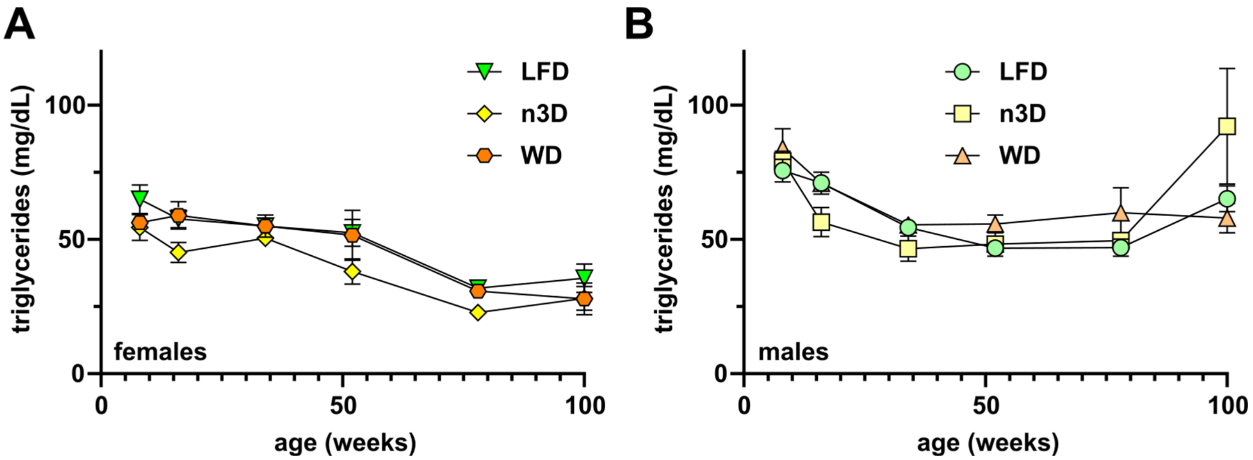

Supplementary Figure 3: Fasting plasma triglyceride levels across age and diet. Plasma was collected from mice following a 6 h fast and plasma triglyceride levels were measured in female **(A)** and male **(B)** mice.

Supplementary Figure 4

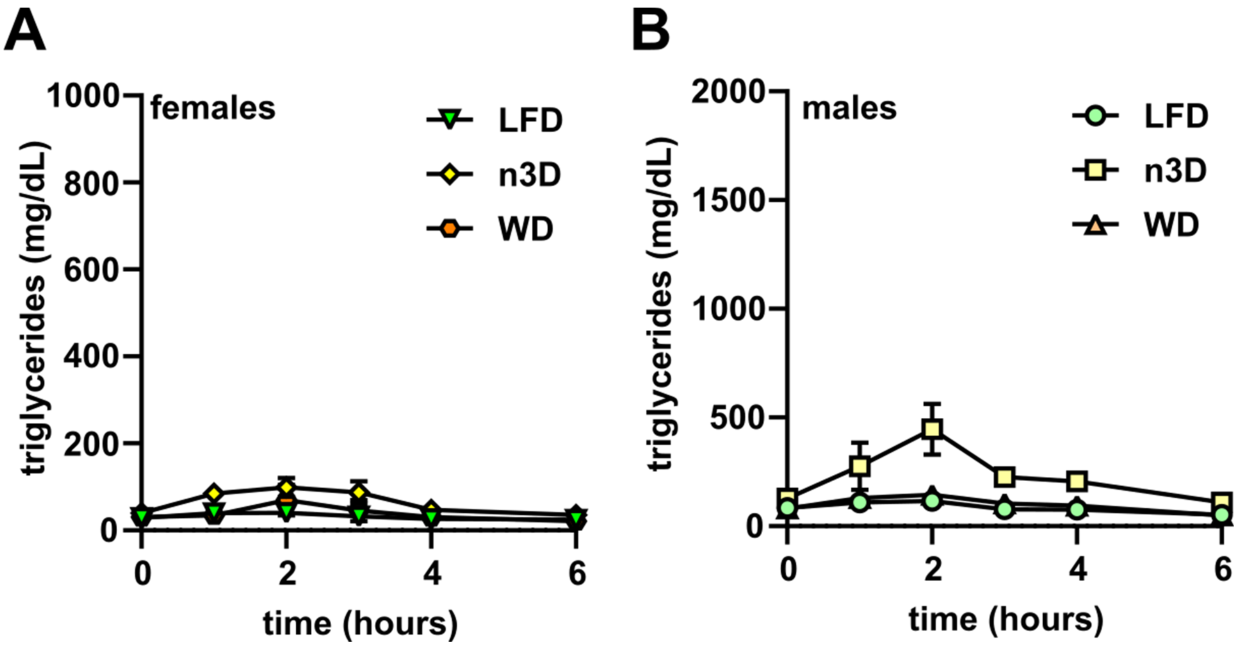

Supplementary Figure 4: Oral fat tolerance tests in old mice. Mice were fasted (12 h) and plasma was collected before and 1, 2, 3, 4, and 6 h after olive oil gavage (10 µl/g body weight). Plasma triglyceride levels were measured at each time point in 101-week old female **(A)** and male **(B)** mice.

Supplementary Figure 5

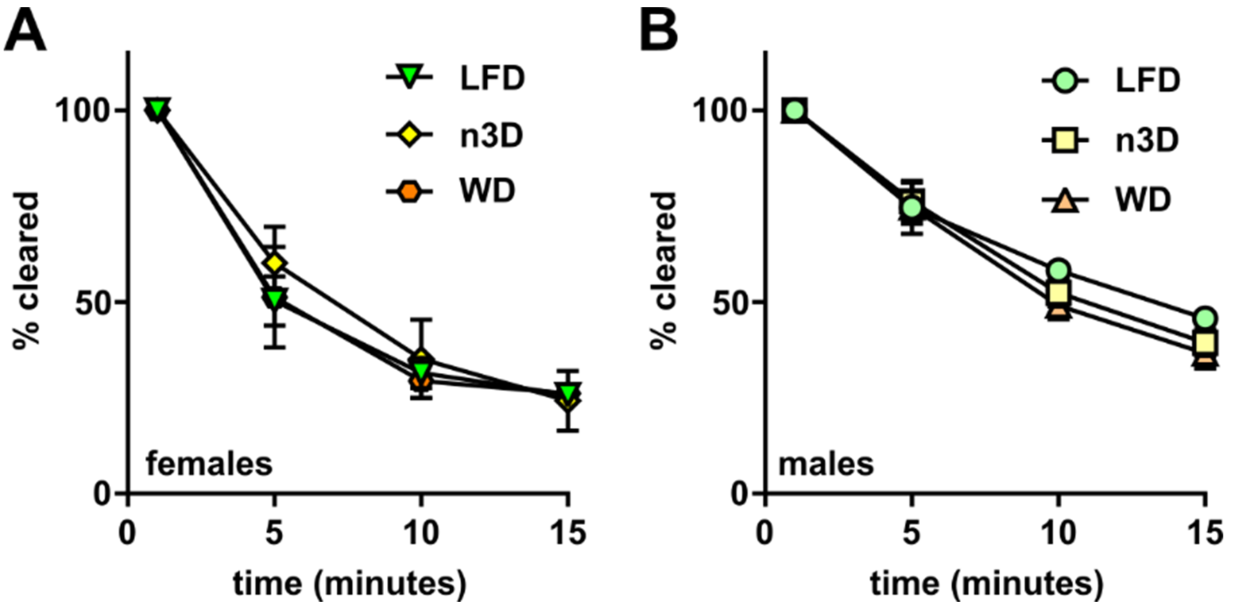

Supplementary Figure 5: Plasma triglyceride clearance in 2-year-old mice. Mice were fasted (6 h) and injected intravenously with ^{3}H-triglyceride containing chylomicrons. Blood was collected after 1, 5, 10, and 15 min after injection to measure clearance of radiolabel from the plasma. Points represent radiolabel remaining in the plasma as a percentage of the 1-min time point (means ± SEM) in 2-year-old female **(A)** and male **(B)** mice.

Supplementary Figure 6

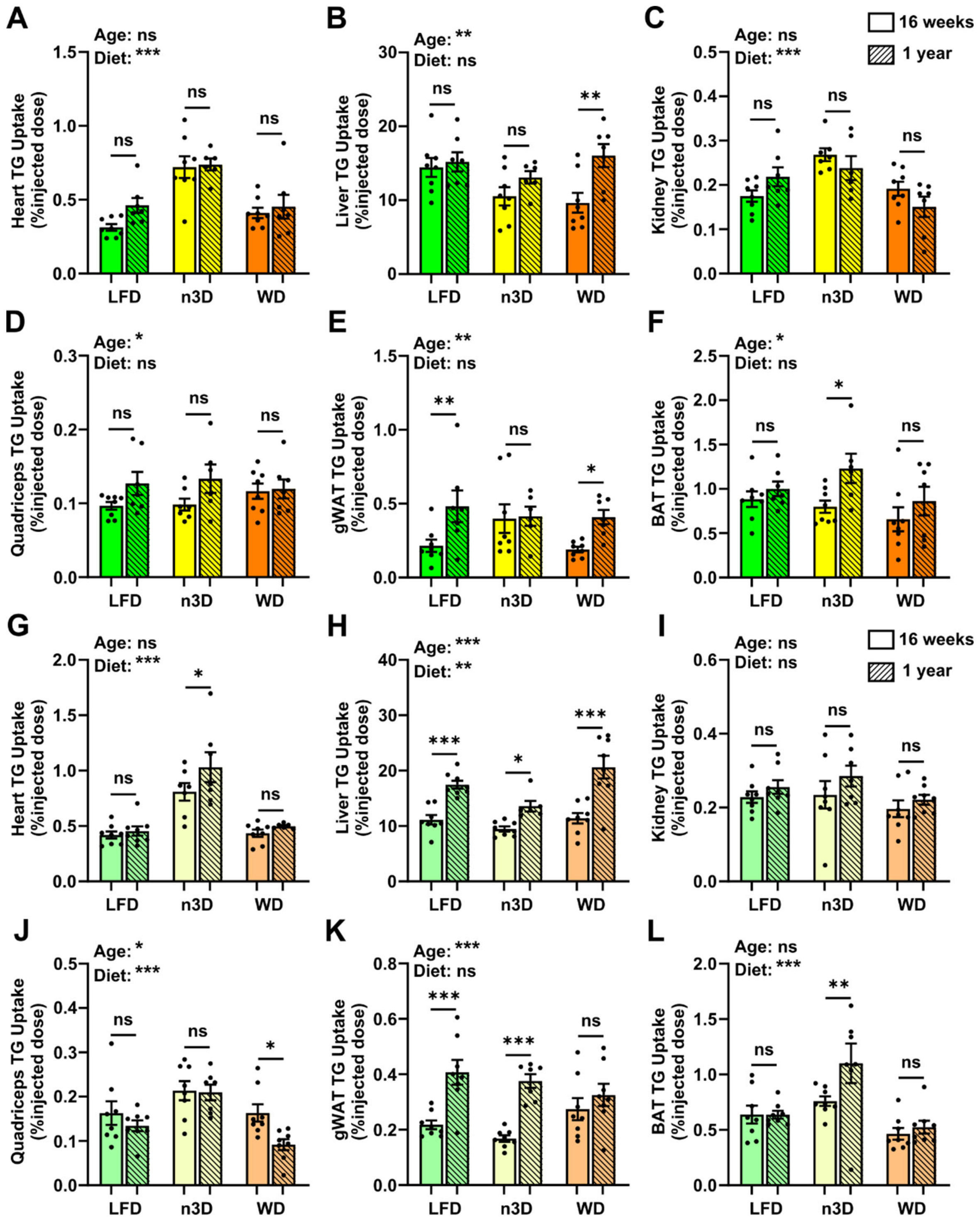

Supplementary Figure 6: Total tissue triglyceride uptake. At 16 weeks or 1 year of age female **(A-F)** and male **(G-L)** mice were fasted (6 h) and injected intravenously with ^{3}H-triglyceride containing chylomicrons. After 15 minutes, tissues were harvested and uptake of radiolabel (% injected dose for total tissue) was measured in heart **(A,G)**, liver **(B,H)**, kidney **(C,I)**, quadriceps muscle **(D,J)**, gonadal white adipose tissue (gWAT)**(E,K)**, and brown adipose (BAT)**(F,L)**. Bars represent mean ± SEM. ns=not significant, *p<0.05, **p<0.01, ***p<0.001 by 2-way ANOVA and subsequent Tukey's multiple comparison test. Complete lists of p values are listed in Supplementary Table 1.

Supplementary Figure 7

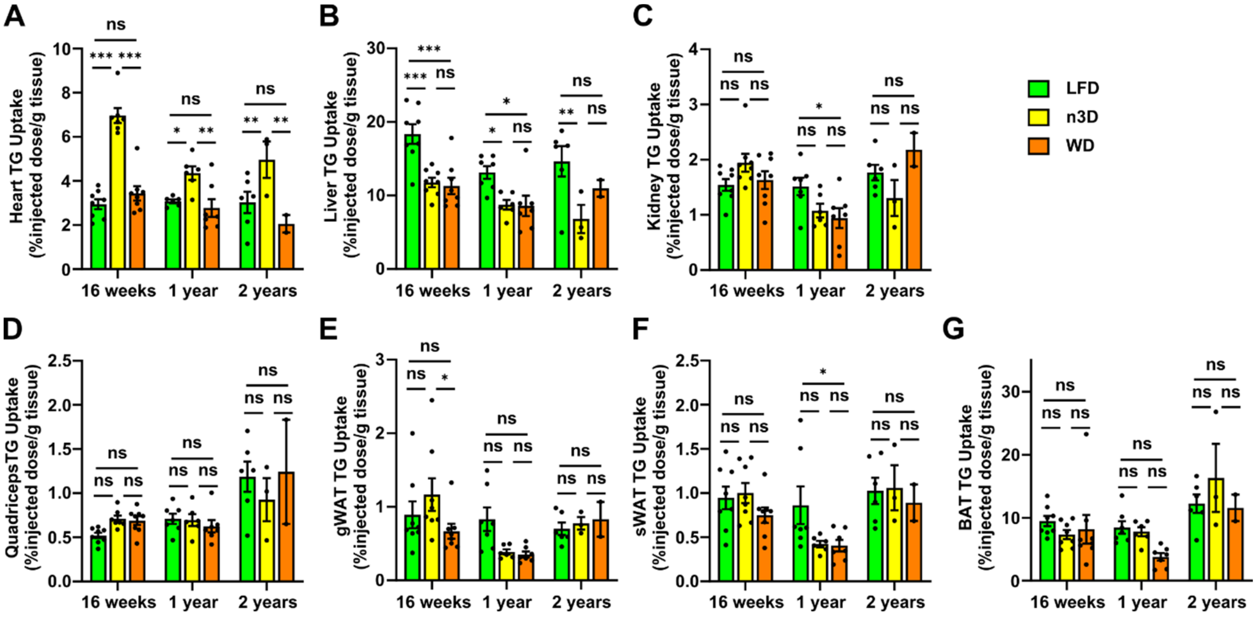

Supplementary Figure 7: Tissue triglyceride uptake in female mice. At 16 weeks, 1 year, or 2 years of age female mice were fasted (6 h) and injected intravenously with ^{3}H-triglyceride containing chylomicrons. After 15 minutes, tissues were harvested and uptake of radiolabel (% injected dose/g tissue) was measured in heart **(A)**, liver **(B)**, kidney **(C)**, quadriceps muscle **(D)**, gonadal white adipose tissue (gWAT)**(E)**, subcutaneous adipose tissue (sWAT)**(F)**, and brown adipose (BAT)**(G)**. Bars represent mean ± SEM. ns=not significant, *p<0.05, **p<0.01, ***p<0.001 by Tukey's multiple comparison test after 2-way ANOVA. Complete lists of p values are listed in Supplementary Table 1.

Supplementary Figure 8

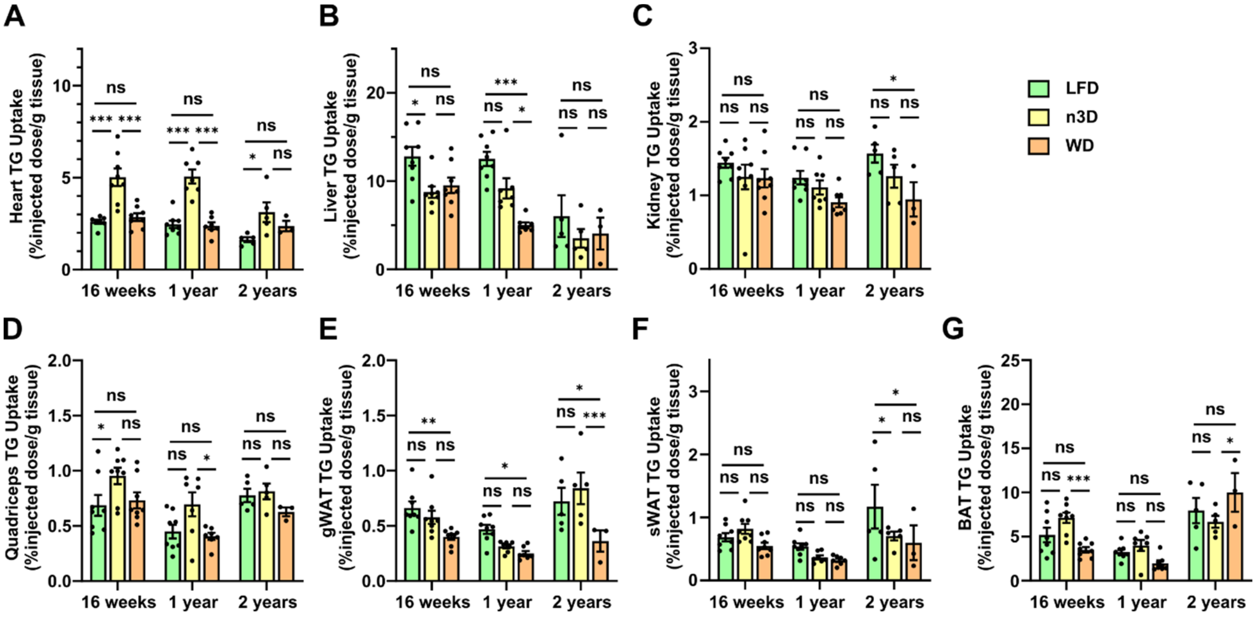

Supplementary Figure 8: Tissue triglyceride uptake in male mice. At 16 weeks, 1 year, or 2 years of age male mice were fasted (6 h) and injected intravenously with ^{3}H-triglyceride containing chylomicrons. After 15 minutes, tissues were harvested and uptake of radiolabel (% injected dose/g tissue) was measured in heart **(A)**, liver **(B)**, kidney **(C)**, quadriceps muscle **(D)**, gonadal white adipose tissue (gWAT)**(E)**, subcutaneous adipose tissue (sWAT)**(F)**, and brown adipose (BAT)**(G)**. Bars represent mean ± SEM. ns=not significant, *p<0.05, **p<0.01, ***p<0.001 by Tukey's multiple comparison test after 2-way ANOVA. Complete lists of p values are listed in Supplementary Table 1.

Supplementary Figure 9

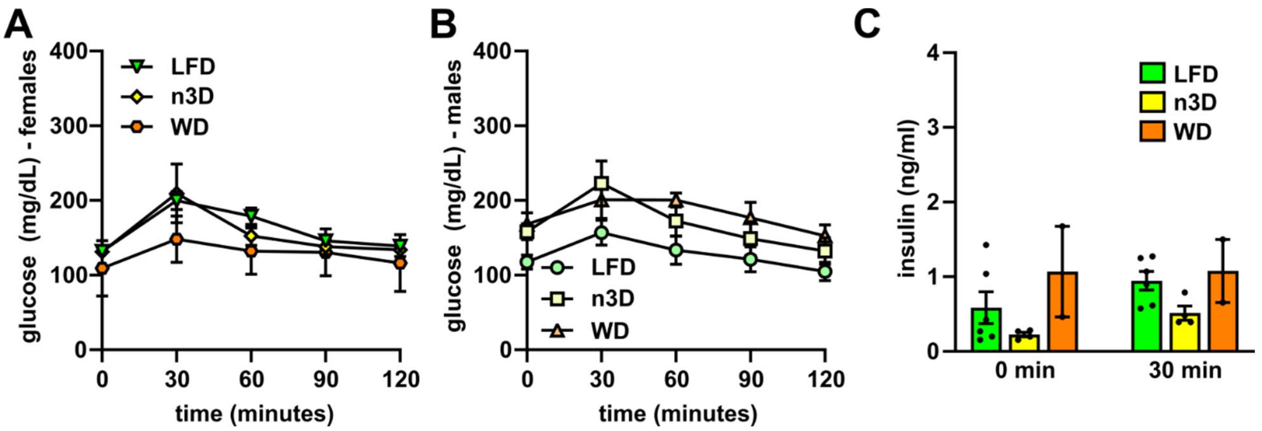

Supplementary Figure 9: Glucose tolerance tests and plasma insulin levels in old mice. **A-B)** Glucose tolerance tests were performed on fasted (6 h) female (A) and male (B) mice at 102 weeks of age. Mice were injected with glucose (1 g/kg) and blood glucose concentrations were measured over 2 h. **C)** Plasma insulin levels before and 30 minutes after glucose injection in 102-week-old female mice.

Supplementary Figure 10

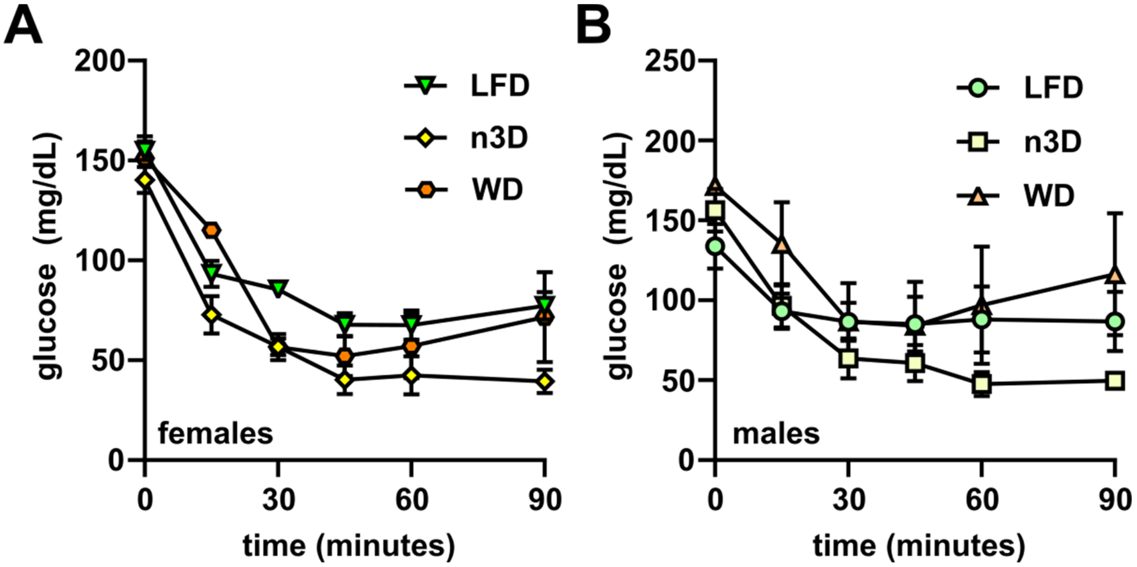

Supplementary Figure 10: Insulin tolerance tests. Insulin tolerance tests were performed in fasted (4 h) female **(A)** and male **(B)** mice at 103 weeks of age. Mice were injected with 0.5 U/mL of human insulin (Humalin-R) and blood glucose concentrations were measured over 90 min. Points represent glucose levels (means ± SEM) at each respective time point.

Supplementary Table 1

Figure 1 statistics

Panel B (All Survival)

	# of animals
LFD	18
n3D	18
WD	18
Survival Log-Rank (Mantel-Cox) test	
	P value
LFD vs n3D	0.409
LFD vs WD	0.003**
n3D vs WD	0.054

Panel C (Female Survival)

	# of animals
LFD	10
n3D	10
WD	10
Survival Log-Rank (Mantel-Cox) test	
	P value
LFD vs n3D	0.400
LFD vs WD	0.013*
n3D vs WD	0.116

Panel D (Male Survival)

	# of animals
LFD	8
n3D	8
WD	8
Survival Log-Rank (Mantel-Cox) test	
	P value
LFD vs n3D	0.861
LFD vs WD	0.149
n3D vs WD	0.236

Figure 2 statistics

Panel A (Female body weights)

	# of animals
LFD	8 (10-2 mice died=8)
n3D	5 (10-5 mice died=5)
WD	5 (10-5 mice died=5)

Panel B (Male body weights)

	# of animals
LFD	8
n3D	7 (8-1 mouse died=7)
WD	5 (8-3 mice died=5)

Panel C (Female food consumption)

	# of cages (animals)
LFD	5 (17)
n3D	5 (15)
WD	7 (16)
	P value
1-way ANOVA	0.001**
Tukey's multiple comparison	
LFD vs n3D	0.262
LFD vs WD	0.001**
n3D vs WD	0.030*

Panel D (Male food consumption)

	# of cages (animals)
LFD	4 (16)
n3D	4 (15)
WD	4 (16)
	P value
1-way ANOVA	0.003**
Tukey's multiple comparison	
LFD vs n3D	0.915
LFD vs WD	0.009**
n3D vs WD	0.003**

Figure 3 statistics

Panel A (Female Lean Mass)

	# of animals
LFD	8 (10-2 mice died=8)
n3D	5 (10-5 mice died=5)
WD	5 (10-5 mice died=5)
	P value
2-way ANOVA	
Age	<0.001***
Diet	<0.001***
Tukey's multiple comparison	
8 weeks (pre-diet)	
LFD vs n3D	0.851
LFD vs WD	0.912
n3D vs WD	0.992
16 weeks	
LFD vs n3D	0.017*
LFD vs WD	0.151
n3D vs WD	0.683
8 months	
LFD vs n3D	0.003**
LFD vs WD	0.545
n3D vs WD	0.093
1 year	
LFD vs n3D	<0.001***
LFD vs WD	0.170
n3D vs WD	0.171
1.5 years	

LFD vs n3D	<0.001***
LFD vs WD	0.001**
n3D vs WD	0.589
LFD	
8 weeks (pre-diet) vs. 16 weeks	0.139
8 weeks (pre-diet) vs. 8 months	<0.001***
8 weeks (pre-diet) vs. 1 year	<0.001***
8 weeks (pre-diet) vs. 1.5 years	<0.001***
16 weeks vs. 8 months	0.010**
16 weeks vs. 1 year	<0.001***
16 weeks vs. 1.5 years	<0.001***
8 months vs. 1 year	0.297
8 months vs. 1.5 years	0.515
1 year vs. 1.5 years	0.996
n3D	
8 weeks (pre-diet) vs. 16 weeks	0.002**
8 weeks (pre-diet) vs. 8 months	<0.001***
8 weeks (pre-diet) vs. 1 year	<0.001***
8 weeks (pre-diet) vs. 1.5 years	<0.001***
16 weeks vs. 8 months	0.016*
16 weeks vs. 1 year	<0.001***
16 weeks vs. 1.5 years	<0.001***
8 months vs. 1 year	0.307
8 months vs. 1.5 years	0.115
1 year vs. 1.5 years	0.986
WD	
8 weeks (pre-diet) vs. 16 weeks	0.017*
8 weeks (pre-diet) vs. 8 months	<0.001***
8 weeks (pre-diet) vs. 1 year	<0.001***
8 weeks (pre-diet) vs. 1.5 years	<0.001***
16 weeks vs. 8 months	0.307
16 weeks vs. 1 year	<0.001***
16 weeks vs. 1.5 years	<0.001***
8 months vs. 1 year	0.178
8 months vs. 1.5 years	0.005**
1 year vs. 1.5 years	0.666

Panel B (Male Lean Mass)

	# of animals
LFD	8
n3D	7 (8-1 mouse died=7)
WD	5 (8-3 mice died=5)
	P value
2-way ANOVA	
Age	<0.001***
Diet	<0.001***
Tukey's multiple comparison	
8 weeks (pre-diet)	
LFD vs n3D	0.725
LFD vs WD	0.537
n3D vs WD	0.205
16 weeks	
LFD vs n3D	0.612

LFD vs WD	0.009**
n3D vs WD	0.095
8 months	
LFD vs n3D	0.660
LFD vs WD	<0.001***
n3D vs WD	0.001**
1 year	
LFD vs n3D	0.188
LFD vs WD	<0.001***
n3D vs WD	0.003**
1.5 years	
LFD vs n3D	<0.001***
LFD vs WD	0.003**
n3D vs WD	0.909
LFD	
8 weeks (pre-diet) vs. 16 weeks	0.010*
8 weeks (pre-diet) vs. 8 months	<0.001***
8 weeks (pre-diet) vs. 1 year	<0.001***
8 weeks (pre-diet) vs. 1.5 years	<0.001***
16 weeks vs. 8 months	0.173
16 weeks vs. 1 year	0.029*
16 weeks vs. 1.5 years	0.106
8 months vs. 1 year	0.944
8 months vs. 1.5 years	0.999
1 year vs. 1.5 years	0.985
n3D	
8 weeks (pre-diet) vs. 16 weeks	<0.001***
8 weeks (pre-diet) vs. 8 months	<0.001***
8 weeks (pre-diet) vs. 1 year	<0.001***
8 weeks (pre-diet) vs. 1.5 years	<0.001***
16 weeks vs. 8 months	0.284
16 weeks vs. 1 year	0.005**
16 weeks vs. 1.5 years	<0.001***
8 months vs. 1 year	0.586
8 months vs. 1.5 years	0.011*
1 year vs. 1.5 years	0.311
WD	
8 weeks (pre-diet) vs. 16 weeks	<0.001***
8 weeks (pre-diet) vs. 8 months	<0.001***
8 weeks (pre-diet) vs. 1 year	<0.001***
8 weeks (pre-diet) vs. 1.5 years	<0.001***
16 weeks vs. 8 months	0.011*
16 weeks vs. 1 year	<0.001***
16 weeks vs. 1.5 years	0.146
8 months vs. 1 year	0.906
8 months vs. 1.5 years	0.857
1 year vs. 1.5 years	0.342

Panel C (Female Fat Mass)

	# of animals
LFD	8 (10-2 mice died=8)
n3D	5 (10-5 mice died=5)
WD	5 (10-5 mice died=5)

	P value
2-way ANOVA	
Age	<0.001***
Diet	<0.001***
Tukey's multiple comparison	
8 weeks (pre-diet)	
LFD vs n3D	0.962
LFD vs WD	0.971
n3D vs WD	0.999
16 weeks	
LFD vs n3D	0.350
LFD vs WD	0.999
n3D vs WD	0.451
8 months	
LFD vs n3D	0.682
LFD vs WD	<0.001***
n3D vs WD	<0.001***
1 year	
LFD vs n3D	0.507
LFD vs WD	<0.001***
n3D vs WD	<0.001***
1.5 years	
LFD vs n3D	0.781
LFD vs WD	<0.001***
n3D vs WD	<0.001***
LFD	
8 weeks (pre-diet) vs. 16 weeks	0.050
8 weeks (pre-diet) vs. 8 months	0.009**
8 weeks (pre-diet) vs. 1 year	<0.001***
8 weeks (pre-diet) vs. 1.5 years	<0.001***
16 weeks vs. 8 months	0.973
16 weeks vs. 1 year	0.024*
16 weeks vs. 1.5 years	0.001**
8 months vs. 1 year	0.111
8 months vs. 1.5 years	0.008**
1 year vs. 1.5 years	0.863
n3D	
8 weeks (pre-diet) vs. 16 weeks	0.755
8 weeks (pre-diet) vs. 8 months	0.199
8 weeks (pre-diet) vs. 1 year	0.002**
8 weeks (pre-diet) vs. 1.5 years	<0.001***
16 weeks vs. 8 months	0.862
16 weeks vs. 1 year	0.067
16 weeks vs. 1.5 years	0.002**
8 months vs. 1 year	0.444
8 months vs. 1.5 years	0.041*
1 year vs. 1.5 years	0.764
WD	
8 weeks (pre-diet) vs. 16 weeks	0.135
8 weeks (pre-diet) vs. 8 months	<0.001***
8 weeks (pre-diet) vs. 1 year	<0.001***
8 weeks (pre-diet) vs. 1.5 years	<0.001***
16 weeks vs. 8 months	<0.001***

16 weeks vs. 1 year	<0.001***
16 weeks vs. 1.5 years	<0.001***
8 months vs. 1 year	<0.001***
8 months vs. 1.5 years	<0.001***
1 year vs. 1.5 years	0.130

Panel D (Male Fat Mass)

	# of animals
LFD	8
n3D	7 (8-1 mouse died=7)
WD	5 (8-3 mice died=5)
	P value
2-way ANOVA	
Age	<0.001***
Diet	<0.001***
Tukey's multiple comparison	
8 weeks (pre-diet)	
LFD vs n3D	0.986
LFD vs WD	0.975
n3D vs WD	0.998
16 weeks	
LFD vs n3D	0.523
LFD vs WD	0.105
n3D vs WD	0.009**
8 months	
LFD vs n3D	0.500
LFD vs WD	<0.001***
n3D vs WD	0.004**
1 year	
LFD vs n3D	0.968
LFD vs WD	<0.001***
n3D vs WD	<0.001***
1.5 years	
LFD vs n3D	0.046*
LFD vs WD	<0.001***
n3D vs WD	0.247
LFD	
8 weeks (pre-diet) vs. 16 weeks	0.468
8 weeks (pre-diet) vs. 8 months	0.009**
8 weeks (pre-diet) vs. 1 year	<0.001***
8 weeks (pre-diet) vs. 1.5 years	0.014*
16 weeks vs. 8 months	0.419
16 weeks vs. 1 year	0.015*
16 weeks vs. 1.5 years	0.506
8 months vs. 1 year	0.578
8 months vs. 1.5 years	>0.9999
1 year vs. 1.5 years	0.489
n3D	
8 weeks (pre-diet) vs. 16 weeks	0.967
8 weeks (pre-diet) vs. 8 months	<0.001***
8 weeks (pre-diet) vs. 1 year	<0.001***
8 weeks (pre-diet) vs. 1.5 years	<0.001***
16 weeks vs. 8 months	0.004**

16 weeks vs. 1 year	<0.001***
16 weeks vs. 1.5 years	<0.001***
8 months vs. 1 year	0.990
8 months vs. 1.5 years	0.840
1 year vs. 1.5 years	0.976
WD	
8 weeks (pre-diet) vs. 16 weeks	0.005**
8 weeks (pre-diet) vs. 8 months	<0.001***
8 weeks (pre-diet) vs. 1 year	<0.001***
8 weeks (pre-diet) vs. 1.5 years	<0.001***
16 weeks vs. 8 months	0.002**
16 weeks vs. 1 year	<0.001***
16 weeks vs. 1.5 years	0.021*
8 months vs. 1 year	0.609
8 months vs. 1.5 years	0.972
1 year vs. 1.5 years	0.284

Figure 4 statistics

Panel A (Plasma TG females)

	# of animals
LFD	8 (10-2 mice died=8)
n3D	5 (10-5 mice died=5)
WD	5 (10-5 mice died=5)
	P value
Mixed effects analysis	
Age	<0.001***
Diet	<0.005**
Tukey's multiple comparison	
8 weeks (pre-diet)	
LFD vs n3D	0.228
LFD vs WD	0.105
n3D vs WD	0.983
16 weeks	
LFD vs n3D	0.126
LFD vs WD	0.985
n3D vs WD	0.218
8 months	
LFD vs n3D	0.741
LFD vs WD	0.584
n3D vs WD	0.984
1 year	
LFD vs n3D	0.034*
LFD vs WD	0.366
n3D vs WD	0.159
1.5 years	
LFD vs n3D	0.064
LFD vs WD	0.953
n3D vs WD	0.224

Panel B (Plasma TG males)

	# of animals
LFD	8

n3D	7 (8-1 mouse died=7)
WD	5 (8-3 mice died=5)
	P value
Mixed effects analysis	
Age	<0.001***
Diet	0.008**
Tukey's multiple comparison	
8 weeks (pre-diet)	
LFD vs n3D	0.939
LFD vs WD	0.152
n3D vs WD	0.193
16 weeks	
LFD vs n3D	0.175
LFD vs WD	0.418
n3D vs WD	0.051
8 months	
LFD vs n3D	0.251
LFD vs WD	0.940
n3D vs WD	0.225
1 year	
LFD vs n3D	>0.999
LFD vs WD	0.158
n3D vs WD	0.314
1.5 years	
LFD vs n3D	0.809
LFD vs WD	0.441
n3D vs WD	0.572

Figure 5 statistics

Panel A (Female FTT 13 weeks)

	# of animals
LFD	8
n3D	8
WD	8
	P-Value
1-Way ANOVA of AUC	0.047*
Tukey's multiple comparison	
LFD vs n3D	0.039*
LFD vs WD	0.584
n3D vs WD	0.252

Panel B (Male FTT 13 weeks)

	# of animals
LFD	8
n3D	8
WD	8
	P-Value
1-Way ANOVA of AUC	0.778
Tukey's multiple comparison	
LFD vs n3D	0.900
LFD vs WD	0.763
n3D vs WD	0.961

Panel C (Female FTT 1 year)

	# of animals
LFD	7 (8-1 mouse died=7)
n3D	6 (8-2 mice died=6)
WD	7 (8-1 mouse died=7)
	P-Value
1-Way ANOVA of AUC	<0.001***
Tukey's multiple comparison	
LFD vs n3D	<0.001***
LFD vs WD	0.961
n3D vs WD	<0.001***

Panel D (Male FTT 1 year)

	# of animals
LFD	7 (8-1 outlier value=7)
n3D	7 (8-1 mouse died=7)
WD	8
	P-Value
1-Way ANOVA of AUC	0.013*
Tukey's multiple comparison	
LFD vs n3D	0.020*
LFD vs WD	0.954
n3D vs WD	0.030*

Panel E (Female FTT 1.5 year)

	# of animals
LFD	8 (10-2 mice died=8)
n3D	8 (10-2 mice died=8)
WD	4 (10-5 mice died-1 outlier value=4)
	P-Value
1-Way ANOVA of AUC	<0.001***
Tukey's multiple comparison	
LFD vs n3D	0.010**
LFD vs WD	0.384
n3D vs WD	0.002**

Panel F (Male FTT 1.5 year)

	# of animals
LFD	8
n3D	7 (8-1 mouse died=7)
WD	5 (8-3 mice died=5)
	P-Value
1-Way ANOVA of AUC	0.054
Tukey's multiple comparison	
LFD vs n3D	0.107
LFD vs WD	0.891
n3D vs WD	0.074

Figure 6 statistics

Panel A (Female 16-week chylomicron clearance)

	# of animals
LFD	8

n3D	8
WD	8
	P value
2-way repeated measures ANOVA	
Diet	0.1332
Time	<0.001***
Tukey's multiple comparison	
5 minutes	
LFD vs n3D	0.007**
LFD vs WD	0.571
n3D vs WD	0.310
10 minutes	
LFD vs n3D	0.169
LFD vs WD	0.866
n3D vs WD	0.625
15 minutes	
LFD vs n3D	0.995
LFD vs WD	0.665
n3D vs WD	0.832

Panel B (Male 16-week chylomicron clearance)

	# of animals
LFD	8
n3D	8
WD	8
	P value
2-way repeated measures ANOVA	
Diet	0.418
Time	<0.001***
Tukey's multiple comparison	
5 minutes	
LFD vs n3D	0.648
LFD vs WD	0.927
n3D vs WD	0.523
10 minutes	
LFD vs n3D	0.522
LFD vs WD	0.778
n3D vs WD	0.341
15 minutes	
LFD vs n3D	0.884
LFD vs WD	0.250
n3D vs WD	0.670

Panel C (Female 1-year chylomicron clearance)

	# of animals
LFD	7 (8-1 mouse died=7)
n3D	6 (8-2 mice died=6)
WD	7 (8-1 mouse died=7)
	P value
2-way repeated measures ANOVA	
Diet	0.444
Time	<0.001**
Tukey's multiple comparison	

5 minutes	
LFD vs n3D	0.946
LFD vs WD	0.799
n3D vs WD	0.519
10 minutes	
LFD vs n3D	0.521
LFD vs WD	0.428
n3D vs WD	0.921
15 minutes	
LFD vs n3D	0.076
LFD vs WD	0.140
n3D vs WD	0.983

Panel D (Male 1-year chylomicron clearance)

	# of animals
LFD	8
n3D	7 (8-1 mouse died=7)
WD	7 (8-1 mouse died=7)
	P value
2-way repeated measures ANOVA	
Diet	<0.001**
Time	<0.001**
Tukey's multiple comparison	
5 minutes	
LFD vs n3D	0.361
LFD vs WD	0.062
n3D vs WD	0.014*
10 minutes	
LFD vs n3D	0.269
LFD vs WD	0.035*
n3D vs WD	0.005**
15 minutes	
LFD vs n3D	0.576
LFD vs WD	<0.001**
n3D vs WD	0.003**

<u>Figure 7 statistics</u>

Panel A (Female Heart TG uptake)

	# of animals
LFD	
16 weeks	8
1 year	6 (8-1 mouse died-1 outlier value=6)
n3D	
16 weeks	7 (8-1 outlier value=7))
1 year	6 (8-2 mice died=6)
WD	
16 weeks	8
1 year	7 (8-1 mouse died=7)
	P value
2-way ANOVA	
Age	<0.001***
Diet	<0.001***

Tukey’s multiple comparison	
16 weeks	
LFD vs n3D	<0.001***
LFD vs WD	0.445
n3D vs WD	<0.001***
1 year	
LFD vs n3D	0.024*
LFD vs WD	0.786
n3D vs WD	0.003**
LFD	
16 weeks vs. 1 year	0.764
n3D	
16 weeks vs. 1 year	<0.001***
WD	
16 weeks vs. 1 year	0.122

Panel B (Female Liver TG uptake)

	# of animals
LFD	
16 weeks	8
1 year	7 (8-1 mouse died=7)
n3D	
16 weeks	8
1 year	6 (8-2 mice died=6)
WD	
16 weeks	8
1 year	7 (8-1 mouse died=7)
	P value
2-way ANOVA	
Age	<0.001***
Diet	<0.001***
Tukey’s multiple comparison	
16 weeks	
LFD vs n3D	<0.001***
LFD vs WD	<0.001***
n3D vs WD	0.942
1 year	
LFD vs n3D	0.025*
LFD vs WD	0.015*
n3D vs WD	0.995
LFD	
16 weeks vs. 1 year	0.001**
n3D	
16 weeks vs. 1 year	0.060
WD	
16 weeks vs. 1 year	0.078

Panel C (Female Kidney TG uptake)

	# of animals
LFD	
16 weeks	7 (8-1 mouse died=7)
1 year	
n3D	8

16 weeks	6 (8-2 mice died=6)
1 year	
WD	8
16 weeks	7 (8-1 mouse died=7)
1 year	7 (8-1 mouse died=7)
	P value
2-way ANOVA	
Age	<0.001***
Diet	0.215
Tukey's multiple comparison	
16 weeks	
LFD vs n3D	0.136
LFD vs WD	0.902
n3D vs WD	0.289
1 year	
LFD vs n3D	0.145
LFD vs WD	0.033*
n3D vs WD	0.827
LFD	
16 weeks vs. 1 year	0.903
n3D	
16 weeks vs. 1 year	<0.001***
WD	
16 weeks vs. 1 year	0.002**

Panel D (Female Quadriceps TG uptake)

	# of animals
LFD	
16 weeks	8
1 year	7 (8-1 mouse died=7)
n3D	
16 weeks	7 (8-1 outlier value=7)
1 year	6 (8-2 mice died=6)
WD	
16 weeks	8
1 year	7 (8-1 mouse died=7)
	P value
2-way ANOVA	
Age	0.395
Diet	0.273
Tukey's multiple comparison	
16 weeks	
LFD vs ω3FD	0.036*
LFD vs WD	0.055
ω3FD vs WD	0.960
1 year	
LFD vs ω3FD	0.985
LFD vs WD	0.508
ω3FD vs WD	0.638
LFD 16 weeks vs 1 year	0.014*
ω3FD 16 weeks vs 1 year	0.869
WD 16 weeks vs 1 year	0.383

Panel E (Female gWAT TG uptake)

	# of animals
LFD	
16 weeks	8
1 year	7 (8-1 mouse died=7)
n3D	
16 weeks	8
1 year	6 (8-2 mice died=6)
WD	
16 weeks	8
1 year	7 (8-1 mouse died=7)
	P value
2-way ANOVA	
Age	0.004**
Diet	0.061
Tukey's multiple comparison	
16 weeks	
LFD vs ω3FD	0.383
LFD vs WD	0.520
ω3FD vs WD	0.050*
1 year	
LFD vs ω3FD	0.137
LFD vs WD	0.084
ω3FD vs WD	0.987
LFD 16 weeks vs 1 year	0.774
ω3FD 16 weeks vs 1 year	0.001**
WD 16 weeks vs 1 year	0.142

Panel F (Female sWAT TG uptake)

	# of animals
LFD	
16 weeks	8
1 year	7 (8-1 mouse died=7)
n3D	
16 weeks	8
1 year	6 (8-2 mice died=6)
WD	
16 weeks	8
1 year	7 (8-1 mouse died=7)
	P value
2-way ANOVA	
Age	0.002**
Diet	0.035*
Tukey's multiple comparison	
16 weeks	
LFD vs ω3FD	0.946
LFD vs WD	0.467
ω3FD vs WD	0.296
1 year	
LFD vs ω3FD	0.058
LFD vs WD	0.037*
ω3FD vs WD	0.995
LFD 16 weeks vs 1 year	0.630

ω3FD 16 weeks vs 1 year	0.003**
WD 16 weeks vs 1 year	0.053

Panel G (Female BAT TG uptake)

	# of animals
LFD	
16 weeks	8
1 year	7 (8-1 mouse died=7)
n3D	
16 weeks	8
1 year	6 (8-2 mice died=6)
WD	
16 weeks	8
1 year	7 (8-1 mouse died=7)
	P value
2-way ANOVA	
Age	0.112
Diet	0.064
Tukey's multiple comparison	
16 weeks	
LFD vs ω3FD	0.415
LFD vs WD	0.722
ω3FD vs WD	0.868
1 year	
LFD vs ω3FD	0.935
LFD vs WD	0.034*
ω3FD vs WD	0.092
LFD 16 weeks vs 1 year	0.557
ω3FD 16 weeks vs 1 year	0.798
WD 16 weeks vs 1 year	0.015*

Figure 8 statistics

Panel A (Male Heart TG uptake)

	# of animals
LFD	
16 weeks	7 (8-outlier value=7)
1 year	8
n3D	
16 weeks	8
1 year	7 (8-1 mouse died=7)
WD	
16 weeks	8
1 year	7 (8-1 mouse died=7)
	P value
2-way ANOVA	
Age	0.445
Diet	<0.001***
Tukey's multiple comparison	
16 weeks	
LFD vs ω3FD	<0.001***
LFD vs WD	0.826
ω3FD vs WD	<0.001***

1 year	
LFD vs ω3FD	<0.001***
LFD vs WD	0.979
ω3FD vs WD	<0.001***
LFD 16 weeks vs 1 year	0.745
ω3FD 16 weeks vs 1 year	0.914
WD 16 weeks vs 1 year	0.271

Panel B (Male Liver TG uptake)

	# of animals
LFD	
16 weeks	8
1 year	8
n3D	
16 weeks	8
1 year	6 (8-1 mouse died-1 outlier value=6)
WD	
16 weeks	8
1 year	7 (8-1 mouse died=7)
	P value
2-way ANOVA	
Age	0.044*
Diet	<0.001***
Tukey's multiple comparison	
16 weeks	
LFD vs ω3FD	0.004**
LFD vs WD	0.024
ω3FD vs WD	0.796
1 year	
LFD vs ω3FD	0.026*
LFD vs WD	<0.001***
ω3FD vs WD	0.006**
LFD 16 weeks vs 1 year	0.809
ω3FD 16 weeks vs 1 year	0.742
WD 16 weeks vs 1 year	<0.001***

Panel C (Male Kidney TG uptake)

	# of animals
LFD	
16 weeks	8
1 year	8
n3D	
16 weeks	8
1 year	7 (8-1 mouse died=7)
WD	
16 weeks	8
1 year	7 (8-1 mouse died=7)
	P value
2-way ANOVA	
Age	0.017*
Diet	0.054
Tukey's multiple comparison	
16 weeks	

LFD vs ω3FD	0.422
LFD vs WD	0.362
ω3FD vs WD	0.993
1 year	
LFD vs ω3FD	0.673
LFD vs WD	0.098
ω3FD vs WD	0.442
LFD 16 weeks vs 1 year	0.193
ω3FD 16 weeks vs 1 year	0.370
WD 16 weeks vs 1 year	0.045*

Panel D (Male Quadriceps TG uptake)

	# of animals
LFD	
16 weeks	8
1 year	8
n3D	
16 weeks	8
1 year	7 (8-1 mouse died=7)
WD	
16 weeks	8
1 year	7 (8-1 mouse died=7)
	P value
2-way ANOVA	
Age	<0.001***
Diet	0.002**
Tukey's multiple comparison	
16 weeks	
LFD vs ω3FD	0.044*
LFD vs WD	0.907
ω3FD vs WD	0.108
1 year	
LFD vs ω3FD	0.079
LFD vs WD	0.920
ω3FD vs WD	0.040*
LFD 16 weeks vs 1 year	0.032*
ω3FD 16 weeks vs 1 year	0.025*
WD 16 weeks vs 1 year	0.005**

Panel E (Male gWAT TG uptake)

	# of animals
LFD	
16 weeks	8
1 year	8
n3D	
16 weeks	8
1 year	7 (8-1 mouse died=7)
WD	
16 weeks	8
1 year	7 (8-1 mouse died=7)
	P value
2-way ANOVA	
Age	<0.001***

Diet	<0.001***
Tukey's multiple comparison	
16 weeks	
LFD vs ω3FD	0.328
LFD vs WD	<0.001***
ω3FD vs WD	0.013*
1 year	
LFD vs ω3FD	0.042*
LFD vs WD	0.003**
ω3FD vs WD	0.586
LFD 16 weeks vs 1 year	0.002**
ω3FD 16 weeks vs 1 year	<0.001***
WD 16 weeks vs 1 year	0.020*

Panel F (Male sWAT TG uptake)

	# of animals
LFD	
16 weeks	8
1 year	8
n3D	
16 weeks	8
1 year	7 (8-1 mouse died=7)
WD	
16 weeks	8
1 year	7 (8-1 mouse died=7)
	P value
2-way ANOVA	
Age	<0.001***
Diet	0.005**
Tukey's multiple comparison	
16 weeks	
LFD vs ω3FD	0.215
LFD vs WD	0.185
ω3FD vs WD	0.003**
1 year	
LFD vs ω3FD	0.090
LFD vs WD	0.025*
ω3FD vs WD	0.852
LFD 16 weeks vs 1 year	0.065
ω3FD 16 weeks vs 1 year	<0.001***
WD 16 weeks vs 1 year	0.007**

Panel G (Male BAT TG uptake)

	# of animals
LFD	
16 weeks	8
1 year	8
n3D	
16 weeks	8
1 year	7 (8-1 mouse died=7)
WD	
16 weeks	8
1 year	7 (8-1 mouse died=7)

	P value
2-way ANOVA	
Age	<0.001***
Diet	<0.001***
Tukey's multiple comparison	
16 weeks	
LFD vs ω3FD	0.033*
LFD vs WD	0.074
ω3FD vs WD	<0.001***
1 year	
LFD vs ω3FD	0.603
LFD vs WD	0.226
ω3FD vs WD	0.037*
LFD 16 weeks vs 1 year	0.013*
ω3FD 16 weeks vs 1 year	<0.001***
WD 16 weeks vs 1 year	0.051

Figure 9 statistics

Panel A (Female GTT 14 weeks)

	# of animals
LFD	8
n3D	8
WD	8
	P-Value
1-Way ANOVA of AUC	<0.001***
Tukey's multiple comparison	
LFD vs ω3FD	0.007**
LFD vs WD	<0.001***
ω3FD vs WD	0.445

Panel B (Female GTT 50 weeks)

	# of animals
LFD	7 (8-1 mouse died=7)
n3D	6 (8-2 mice died=6)
WD	7 (8-1 mouse died=7)
	P-Value
1-Way ANOVA of AUC	0.296
Tukey's multiple comparison	
LFD vs ω3FD	0.753
LFD vs WD	0.266
ω3FD vs WD	0.690

Panel C (Female GTT 1.5 years)

	# of animals
LFD	8 (10-2 mice died=8)
n3D	8 (10-2 mice died=8)
WD	5 (10-5 mice died=5)
	P-Value
1-Way ANOVA of AUC	0.057
Tukey's multiple comparison	
LFD vs ω3FD	0.490
LFD vs WD	0.046*

ω3FD vs WD	0.281

Panel D (Male GTT 14 weeks)

	# of animals
LFD	8
n3D	8
WD	8
	P-Value
1-Way ANOVA of AUC	0.001**
Tukey's multiple comparison	
LFD vs ω3FD	0.623
LFD vs WD	0.001**
ω3FD vs WD	0.012*

Panel E (Male GTT 50 weeks)

	# of animals
LFD	8
n3D	7 (8-1 mouse died=7)
WD	8
	P-Value
1-Way ANOVA of AUC	0.139
Tukey's multiple comparison	
LFD vs ω3FD	0.518
LFD vs WD	0.580
ω3FD vs WD	0.118

Panel F (Male GTT 1.5 years)

	# of animals
LFD	8
n3D	7 (8-1 mouse died=7)
WD	5 (8-3 mice died=5)
	P-Value
1-Way ANOVA of AUC	0.038*
Tukey's multiple comparison	
LFD vs ω3FD	0.325
LFD vs WD	0.292
ω3FD vs WD	0.030*

Panel G (female insulin 14 weeks)

	# of animals
LFD	8
n3D	8
WD	7
	P value
2-way ANOVA	
Time	<0.001***
Diet	0.004**
Tukey's multiple comparison	
0 minutes	
LFD vs ω3FD	0.036*
LFD vs WD	0.999
ω3FD vs WD	0.050*
30 minutes	

LFD vs ω3FD	0.011*
LFD vs WD	0.778
ω3FD vs WD	0.002**
LFD 0 vs 30 min	0.026*
ω3FD 0 vs 30 min	0.102
WD 0 vs 30 min	0.004**

Panel H (female insulin 50 weeks)

	# of animals
LFD	7 (8-1 mouse died=7)
n3D	6 (8-2 mice died=6)
WD	7 (8-1 mouse died=7)
	P value
2-way ANOVA	
Time	<0.001***
Diet	0.0016
Tukey's multiple comparison	
0 minutes	
LFD vs ω3FD	0.071
LFD vs WD	0.184
ω3FD vs WD	<0.001***
30 minutes	
LFD vs ω3FD	0.270
LFD vs WD	0.020*
ω3FD vs WD	<0.001***
LFD 0 vs 30 min	0.017*
ω3FD 0 vs 30 min	<0.001***
WD 0 vs 30 min	<0.001***

Panel I (female insulin 1.5 years)

	# of animals
LFD	8 (10-2 mice died=8)
n3D	8 (10-2 mice died=8)
WD	5 (10-5 mice died=5)
	P value
2-way ANOVA	
Time	<0.001***
Diet	<0.001***
Tukey's multiple comparison	
0 minutes	
LFD vs ω3FD	0.209
LFD vs WD	0.019*
ω3FD vs WD	<0.001***
30 minutes	
LFD vs ω3FD	0.520
LFD vs WD	<0.001***
ω3FD vs WD	<0.001***
LFD 0 vs 30 min	0.008**
ω3FD 0 vs 30 min	<0.001***
WD 0 vs 30 min	<0.001***

Figure 10 statistics

Panel A (Female ITT 15 weeks)

	# of animals
LFD	8
n3D	8
WD	8
	P-Value
1-Way ANOVA of AUC	0.177
Tukey's multiple comparison	
LFD vs ω3FD	0.793
LFD vs WD	0.434
ω3FD vs WD	0.161

Panel B (Female ITT 51 weeks)

	# of animals
LFD	7 (8-1 mouse died=7)
n3D	6 (8-2 mice died=6)
WD	7 (8-1 mouse died=7)
	P-Value
1-Way ANOVA of AUC	0.282
Tukey's multiple comparison	
LFD vs ω3FD	0.969
LFD vs WD	0.291
ω3FD vs WD	0.373

Panel C (Female ITT 1.5 years)

	# of animals
LFD	5 (10-2 mice died-3 mice didn't finish=5)
n3D	2 (10-2 mice died-6 mice didn't finish=2)
WD	5 (10-5 mice died=5)
	P-Value
1-Way ANOVA of AUC	0.627
Tukey's multiple comparison	
LFD vs ω3FD	0.967
LFD vs WD	0.714
ω3FD vs WD	0.684

Panel D (Male ITT 15 weeks)

	# of animals
LFD	8
n3D	8
WD	8
	P-Value
1-Way ANOVA of AUC	<0.001***
Tukey's multiple comparison	
LFD vs ω3FD	0.040*
LFD vs WD	0.186
ω3FD vs WD	<0.001***

Panel E (Male ITT 51 weeks)

	# of animals
LFD	8

n3D	7 (8-1 mouse died=7)
WD	8
	P-Value
1-Way ANOVA of AUC	0.025*
Tukey's multiple comparison	
LFD vs ω3FD	0.283
LFD vs WD	0.324
ω3FD vs WD	0.019*

Panel F (Male ITT 1.5 years)

# of animal	
LFD	8
n3D	6 (8-1 mouse died-1 outlier value=6)
WD	5 (8-3 mice died=5)
	P-Value
1-Way ANOVA of AUC	0.272
Tukey's multiple comparison	
LFD vs ω3FD	0.994
LFD vs WD	0.320
ω3FD vs WD	0.318

Supplemental Figure 1 statistics

Panel A (Female body weights)

	# of animals
LFD	10 at start
n3D	10 at start
WD	10 at start

Panel B (Male body weights)

	# of animals
LFD	8 at start
n3D	8 at start
WD	8 at start

Supplemental Figure 2 statistics

Panel A (Female Lean Mass)

	# of animals
LFD	
8 weeks (pre-diet)	26
16 weeks	26
8 months	16 (18-2 mice died=16)
1 year	15 (18-3 mice died=15)
1.5 year	8 (10-2 mice died=8)
2 years	6 (10-4 mice died=6)
n3D	
8 weeks (pre-diet)	25 (26-1 outlier value)
16 weeks	26
8 months	15 (18-3 mice died=15)
1 year	14 (18-4 mice died=14)
1.5 year	7 (10-3 mice died=7)

2 years	4 (10-6 mice died=4)
WD	
8 weeks (pre-diet)	26
16 weeks	26
8 months	17 (18-1 mouse died=17
1 year	15 (18-3 mice died=15)
1.5 year	5 (10-5 mice died=5)
2 years	2 (10-8 mice died=2)
	P value
2-way ANOVA	
Age	<0.001***
Diet	<0.001***
Tukey's multiple comparison	
8 weeks (pre-diet)	
LFD vs n3D	0.684
LFD vs WD	0.998
n3D vs WD	0.718
16 weeks	
LFD vs n3D	<0.001***
LFD vs WD	0.003**
n3D vs WD	0.772
8 months	
LFD vs n3D	<0.001***
LFD vs WD	0.004**
n3D vs WD	0.002**
1 year	
LFD vs n3D	<0.001***
LFD vs WD	0.002**
n3D vs WD	0.017*
1.5 years	
LFD vs n3D	<0.001***
LFD vs WD	<0.001***
n3D vs WD	0.465
2 years	
LFD vs n3D	<0.001***
LFD vs WD	<0.001***
n3D vs WD	0.390
LFD	
8 weeks (pre-diet) vs. 16 weeks	0.010**
8 weeks (pre-diet) vs. 8 months	<0.001***
8 weeks (pre-diet) vs. 1 year	<0.001***
8 weeks (pre-diet) vs. 1.5 years	<0.001***
8 weeks (pre-diet) vs. 2 years	<0.001***
16 weeks vs. 8 months	<0.001***
16 weeks vs. 1 year	<0.001***
16 weeks vs. 1.5 years	<0.001***
16 weeks vs. 2 years	<0.001***
8 months vs. 1 year	0.320
8 months vs. 1.5 years	0.617
8 months vs. 2 years	0.623
1 year vs. 1.5 years	>0.999
1 year vs. 2 years	>0.999
1.5 years vs. 2 years	>0.999

n3D	
8 weeks (pre-diet) vs. 16 weeks	<0.001***
8 weeks (pre-diet) vs. 8 months	<0.001***
8 weeks (pre-diet) vs. 1 year	<0.001***
8 weeks (pre-diet) vs. 1.5 years	<0.001***
8 weeks (pre-diet) vs. 2 years	<0.001***
16 weeks vs. 8 months	<0.001***
16 weeks vs. 1 year	<0.001***
16 weeks vs. 1.5 years	<0.001***
16 weeks vs. 2 years	<0.001***
8 months vs. 1 year	0.457
8 months vs. 1.5 years	0.081
8 months vs. 2 years	0.013*
1 year vs. 1.5 years	0.837
1 year vs. 2 years	0.282
1.5 years vs. 2 years	0.908
WD	
8 weeks (pre-diet) vs. 16 weeks	<0.001***
8 weeks (pre-diet) vs. 8 months	<0.001***
8 weeks (pre-diet) vs. 1 year	<0.001***
8 weeks (pre-diet) vs. 1.5 years	<0.001***
8 weeks (pre-diet) vs. 2 years	<0.001***
16 weeks vs. 8 months	<0.001***
16 weeks vs. 1 year	<0.001***
16 weeks vs. 1.5 years	<0.001***
16 weeks vs. 2 years	<0.001***
8 months vs. 1 year	0.127
8 months vs. 1.5 years	0.007**
8 months vs. 2 years	<0.001***
1 year vs. 1.5 years	0.520
1 year vs. 2 years	<0.001***
1.5 years vs. 2 years	0.040*

Panel B (Male Lean Mass)

	# of animals
LFD	
8 weeks (pre-diet)	24
16 weeks	24
8 months	16
1 year	16
1.5 year	8
2 years	6 (8-2 mice died=6)
n3D	
8 weeks (pre-diet)	24
16 weeks	24
8 months	14 (16-1 mouse died-1 mouse not measured=14)
1 year	15 (16-1 mouse died)
1.5 year	7 (8-1 mouse died=7)
2 years	5 (8-3 mice died=5)
WD	
8 weeks (pre-diet)	24
16 weeks	24

8 months	16
1 year	14 (16-2 mice not measured=14)
1.5 year	5 (8-3 mice died=5)
2 years	3 (8-5 mice died=3)
	P value
2-way ANOVA	
Age	<0.001***
Diet	<0.001***
Tukey's multiple comparison	
8 weeks (pre-diet)	
LFD vs n3D	0.648
LFD vs WD	0.946
n3D vs WD	0.451
16 weeks	
LFD vs n3D	0.464
LFD vs WD	0.044*
n3D vs WD	0.439
8 months	
LFD vs n3D	0.182
LFD vs WD	<0.001***
n3D vs WD	0.017*
1 year	
LFD vs n3D	0.006**
LFD vs WD	<0.001***
n3D vs WD	0.008**
1.5 years	
LFD vs n3D	<0.001***
LFD vs WD	0.003**
n3D vs WD	0.917
2 years	
LFD vs n3D	<0.001***
LFD vs WD	0.002**
n3D vs WD	0.993
LFD	
8 weeks (pre-diet) vs. 16 weeks	<0.001***
8 weeks (pre-diet) vs. 8 months	<0.001***
8 weeks (pre-diet) vs. 1 year	<0.001***
8 weeks (pre-diet) vs. 1.5 years	<0.001***
8 weeks (pre-diet) vs. 2 years	<0.001***
16 weeks vs. 8 months	0.010**
16 weeks vs. 1 year	<0.001***
16 weeks vs. 1.5 years	0.065
16 weeks vs. 2 years	0.675
8 months vs. 1 year	0.990
8 months vs. 1.5 years	>0.999
8 months vs. 2 years	0.950
1 year vs. 1.5 years	0.998
1 year vs. 2 years	0.767
1.5 years vs. 2 years	0.959
n3D	
8 weeks (pre-diet) vs. 16 weeks	<0.001***
8 weeks (pre-diet) vs. 8 months	<0.001***
8 weeks (pre-diet) vs. 1 year	<0.001***

8 weeks (pre-diet) vs. 1.5 years	<0.001***
8 weeks (pre-diet) vs. 2 years	<0.001***
16 weeks vs. 8 months	<0.001***
16 weeks vs. 1 year	<0.001***
16 weeks vs. 1.5 years	<0.001***
16 weeks vs. 2 years	<0.001***
8 months vs. 1 year	0.453
8 months vs. 1.5 years	0.019*
8 months vs. 2 years	0.070
1 year vs. 1.5 years	0.489
1 year vs. 2 years	0.692
1.5 years vs. 2 years	>0.999
WD	
8 weeks (pre-diet) vs. 16 weeks	<0.001***
8 weeks (pre-diet) vs. 8 months	<0.001***
8 weeks (pre-diet) vs. 1 year	<0.001***
8 weeks (pre-diet) vs. 1.5 years	<0.001***
8 weeks (pre-diet) vs. 2 years	<0.001***
16 weeks vs. 8 months	<0.001***
16 weeks vs. 1 year	<0.001***
16 weeks vs. 1.5 years	<0.001***
16 weeks vs. 2 years	0.002**
8 months vs. 1 year	0.266
8 months vs. 1.5 years	0.997
8 months vs. 2 years	0.995
1 year vs. 1.5 years	0.901
1 year vs. 2 years	0.980
1.5 years vs. 2 years	>0.999

Panel C (Female Fat Mass)

	# of animals
LFD	
8 weeks (pre-diet)	26
16 weeks	26
8 months	16 (18-2 mice died=16)
1 year	15 (18-3 mice died=15)
1.5 year	8 (10-2 mice died=8)
2 years	6 (10-4 mice died=6)
n3D	
8 weeks (pre-diet)	26
16 weeks	26
8 months	15 (18-3 mice died=15)
1 year	14 (18-4 mice died=14)
1.5 year	7 (10-3 mice died=7)
2 years	4 (10-6 mice died=4)
WD	
8 weeks (pre-diet)	26
16 weeks	24 (26-2 outlier values)
8 months	17 (18-1 mouse died=17)
1 year	15 (18-3 mice died=15)
1.5 year	5 (10-5 mice died=5)
2 years	2 (10-8 mice died=2)
	P value

2-way ANOVA	
Age	<0.001***
Diet	<0.001***
Tukey's multiple comparison	
8 weeks (pre-diet)	
LFD vs n3D	0.974
LFD vs WD	0.993
n3D vs WD	0.994
16 weeks	
LFD vs n3D	0.225
LFD vs WD	0.937
n3D vs WD	0.409
8 months	
LFD vs n3D	0.491
LFD vs WD	<0.001***
n3D vs WD	<0.001***
1 year	
LFD vs n3D	0.397
LFD vs WD	<0.001***
n3D vs WD	<0.001***
1.5 years	
LFD vs n3D	0.997
LFD vs WD	<0.001***
n3D vs WD	<0.001***
2 years	
LFD vs n3D	0.042*
LFD vs WD	0.401
n3D vs WD	0.825
LFD	
8 weeks (pre-diet) vs. 16 weeks	<0.001***
8 weeks (pre-diet) vs. 8 months	<0.001***
8 weeks (pre-diet) vs. 1 year	<0.001***
8 weeks (pre-diet) vs. 1.5 years	<0.001***
8 weeks (pre-diet) vs. 2 years	<0.001***
16 weeks vs. 8 months	0.971
16 weeks vs. 1 year	<0.001***
16 weeks vs. 1.5 years	<0.001***
16 weeks vs. 2 years	0.026*
8 months vs. 1 year	0.004**
8 months vs. 1.5 years	0.001**
8 months vs. 2 years	0.154
1 year vs. 1.5 years	0.949
1 year vs. 2 years	0.999
1.5 years vs. 2 years	0.911
n3D	
8 weeks (pre-diet) vs. 16 weeks	0.028*
8 weeks (pre-diet) vs. 8 months	<0.001***
8 weeks (pre-diet) vs. 1 year	<0.001***
8 weeks (pre-diet) vs. 1.5 years	<0.001***
8 weeks (pre-diet) vs. 2 years	0.271
16 weeks vs. 8 months	0.009**
16 weeks vs. 1 year	<0.001***
16 weeks vs. 1.5 years	<0.001***

16 weeks vs. 2 years	0.994
8 months vs. 1 year	0.003**
8 months vs. 1.5 years	0.068
8 months vs. 2 years	0.692
1 year vs. 1.5 years	>0.999
1 year vs. 2 years	0.002**
1.5 years vs. 2 years	0.013*
WD	
8 weeks (pre-diet) vs. 16 weeks	<0.001***
8 weeks (pre-diet) vs. 8 months	<0.001***
8 weeks (pre-diet) vs. 1 year	<0.001***
8 weeks (pre-diet) vs. 1.5 years	<0.001***
8 weeks (pre-diet) vs. 2 years	0.230
16 weeks vs. 8 months	<0.001***
16 weeks vs. 1 year	<0.001***
16 weeks vs. 1.5 years	<0.001***
16 weeks vs. 2 years	0.991
8 months vs. 1 year	<0.001***
8 months vs. 1.5 years	<0.001***
8 months vs. 2 years	<0.001***
1 year vs. 1.5 years	<0.001***
1 year vs. 2 years	<0.001***
1.5 years vs. 2 years	<0.001***

Panel D (Male Fat Mass)

	# of animals
LFD	
8 weeks (pre-diet)	24
16 weeks	23 (24-1 outlier value=23)
8 months	16
1 year	16
1.5 year	8
2 years	6 (8-2 mice died=6)
n3D	
8 weeks (pre-diet)	24
16 weeks	24
8 months	14 (16-1 mouse died-1 mouse not measured=14)
1 year	15 (16-1 mouse died)
1.5 year	7 (8-1 mouse died=7)
2 years	5 (8-3 mice died=5)
WD	
8 weeks (pre-diet)	24
16 weeks	24
8 months	16
1 year	14 (16-2 mice not measured)
1.5 year	5 (8-3 mice died=5)
2 years	3 (8-5 mice died=3)
	P value
2-way ANOVA	
Age	<0.001***
Diet	<0.001***
Tukey’s multiple comparison	

8 weeks (pre-diet)	
LFD vs n3D	0.996
LFD vs WD	0.997
n3D vs WD	>0.999
16 weeks	
LFD vs n3D	0.399
LFD vs WD	<0.001***
n3D vs WD	<0.001***
8 months	
LFD vs n3D	0.023*
LFD vs WD	<0.001***
n3D vs WD	<0.001***
1 year	
LFD vs n3D	0.586
LFD vs WD	<0.001***
n3D vs WD	<0.001***
1.5 years	
LFD vs n3D	0.016*
LFD vs WD	<0.001***
n3D vs WD	0.154
2 years	
LFD vs n3D	<0.001***
LFD vs WD	<0.001***
n3D vs WD	<0.001***
LFD	
8 weeks (pre-diet) vs. 16 weeks	0.057
8 weeks (pre-diet) vs. 8 months	<0.001***
8 weeks (pre-diet) vs. 1 year	<0.001***
8 weeks (pre-diet) vs. 1.5 years	<0.001***
8 weeks (pre-diet) vs. 2 years	0.253
16 weeks vs. 8 months	<0.001***
16 weeks vs. 1 year	<0.001***
16 weeks vs. 1.5 years	0.084
16 weeks vs. 2 years	0.999
8 months vs. 1 year	0.197
8 months vs. 1.5 years	0.983
8 months vs. 2 years	0.111
1 year vs. 1.5 years	0.109
1 year vs. 2 years	<0.001***
1.5 years vs. 2 years	0.521
n3D	
8 weeks (pre-diet) vs. 16 weeks	0.578
8 weeks (pre-diet) vs. 8 months	<0.001***
8 weeks (pre-diet) vs. 1 year	<0.001***
8 weeks (pre-diet) vs. 1.5 years	<0.001***
8 weeks (pre-diet) vs. 2 years	<0.001***
16 weeks vs. 8 months	<0.001***
16 weeks vs. 1 year	<0.001***
16 weeks vs. 1.5 years	<0.001***
16 weeks vs. 2 years	<0.001***
8 months vs. 1 year	0.995
8 months vs. 1.5 years	0.999
8 months vs. 2 years	0.027*

1 year vs. 1.5 years	>0.9999
1 year vs. 2 years	0.072
1.5 years vs. 2 years	0.144
WD	
8 weeks (pre-diet) vs. 16 weeks	<0.001***
8 weeks (pre-diet) vs. 8 months	<0.001***
8 weeks (pre-diet) vs. 1 year	<0.001***
8 weeks (pre-diet) vs. 1.5 years	<0.001***
8 weeks (pre-diet) vs. 2 years	<0.001***
16 weeks vs. 8 months	<0.001***
16 weeks vs. 1 year	<0.001***
16 weeks vs. 1.5 years	<0.001***
16 weeks vs. 2 years	<0.001***
8 months vs. 1 year	0.003**
8 months vs. 1.5 years	0.817
8 months vs. 2 years	<0.001***
1 year vs. 1.5 years	0.002**
1 year vs. 2 years	0.098
1.5 years vs. 2 years	<0.001***

Supplementary Figure 3 statistics

Panel A (Plasma TG females)

	# of animals
LFD	
8 weeks (pre-diet)	10
16 weeks	10
8 months	9 (10-1 mouse died=9)
1 year	8 (10-2 mice died=8)
1.5 year	8 (10-2 mice died=8)
2 years	6 (10-4 mice died=6)
n3D	
8 weeks (pre-diet)	10
16 weeks	10
8 months	9 (10-1 mouse died=9)
1 year	8 (10-2 mice died=8)
1.5 year	8 (10-2 mice died=8)
2 years	4 (10-6 mice died=4)
WD	
8 weeks (pre-diet)	10
16 weeks	10
8 months	10
1 year	7 (10-2 mice died-1 outlier value=7)
1.5 year	5 (10-5 mice died=5)
2 years	2 (10-8 mice died=2)

Panel B (Plasma TG males)

	# of animals
LFD	
8 weeks (pre-diet)	8
16 weeks	8
8 months	8
1 year	8

1.5 year	8
2 years	6 (8-2 mice died=6)
n3D	
8 weeks (pre-diet)	8
16 weeks	8
8 months	7 (8-1 sample lost=7)
1 year	8
1.5 year	7 (8-1 mouse died=7)
2 years	5 (8-3 mice died=5)
WD	
8 weeks (pre-diet)	8
16 weeks	8
8 months	8
1 year	7 (8-1 sample lost=7)
1.5 year	5 (8-3 mice died=5)
2 years	3 (8-5 mice died=3)

Supplementary Figure 4 statistics

Panel A (Female FTT 2 years)

	# of animals
LFD	6 (10-4 mice died=6)
n3D	4 (10-6 mice died=4)
WD	2 (10-8 mice died=2)

Panel B (Male FTT 2 years)

	# of animals
LFD	6 (8-2 mice died=6)
n3D	5 (8-3 mice died=5)
WD	3 (8-5 mice died=3)

Supplementary Figure 5 statistics

Panel A (Female 2-year chylomicron clearance)

	# of animals
LFD	6 (10-4 mice died=6)
n3D	3 (10-7 mice died=3)
WD	0 (10-10 mice died=0)

Panel B (Male 2-year chylomicron clearance)

	# of animals
LFD	5 (8-3 mice died=5)
n3D	5 (8-3 mice died=5)
WD	3 (8-5 mice died=3)

Supplementary Figure 6 statistics

Panel A (Female Heart TG uptake)

	# of animals
LFD	
16 weeks	8
1 year	7 (8-1 mouse died=7)
n3D	

16 weeks	8
1 year	6 (8-2 mice died=6)
WD	
16 weeks	8
1 year	7 (8-1 mouse died=7)
	P value
2-way ANOVA	
Age	0.129
Diet	<0.001***
Tukey's multiple comparison	
16 weeks	
LFD vs n3D	<0.001***
LFD vs WD	0.390
n3D vs WD	<0.001***
1 year	
LFD vs n3D	0.005**
LFD vs WD	0.993
n3D vs WD	0.004**
LFD	
16 weeks vs. 1 year	0.059
n3D	
16 weeks vs. 1 year	0.826
WD	
16 weeks vs. 1 year	0.586

Panel B (Female Liver TG uptake)

	# of animals
LFD	
16 weeks	8
1 year	7 (8-1 mouse died=7)
n3D	
16 weeks	8
1 year	6 (8-2 mice died=6)
WD	
16 weeks	8
1 year	7 (8-1 mouse died=7)
	P value
2-way ANOVA	
Age	0.004**
Diet	0.079
Tukey's multiple comparison	
16 weeks	
LFD vs n3D	0.082
LFD vs WD	0.026*
n3D vs WD	0.869
1 year	
LFD vs n3D	0.536
LFD vs WD	0.896
n3D vs WD	0.301
LFD	
16 weeks vs. 1 year	0.680
n3D	
16 weeks vs. 1 year	0.188

WD	
16 weeks vs. 1 year	0.001**

Panel C (Female Kidney TG uptake)

	# of animals
LFD	
16 weeks	8
1 year	7 (8-1 mouse died=7)
n3D	
16 weeks	7 (8-1 outlier value=7)
1 year	6 (8-2 mice died=6)
WD	
16 weeks	8
1 year	7 (8-1 mouse died=7)
	P value
2-way ANOVA	
Age	0.562
Diet	<0.001***
Tukey’s multiple comparison	
16 weeks	
LFD vs n3D	0.003**
LFD vs WD	0.794
n3D vs WD	0.016*
1 year	
LFD vs n3D	0.769
LFD vs WD	0.043
n3D vs WD	0.010*
LFD	
16 weeks vs. 1 year	0.105
n3D	
16 weeks vs. 1 year	0.293
WD	
16 weeks vs. 1 year	0.129

Panel D (Female Quadriceps TG uptake)

	# of animals
LFD	
16 weeks	8
1 year	7 (8-1 mouse died=7)
n3D	
16 weeks	7 (8-1 outlier value=7)
1 year	6 (8-2 mice died=6)
WD	
16 weeks	8
1 year	7 (8-1 mouse died=7)
	P value
2-way ANOVA	
Age	0.027*
Diet	0.870
Tukey’s multiple comparison	
16 weeks	
LFD vs n3D	0.994
LFD vs WD	0.446

n3D vs WD	0.530
1 year	
LFD vs n3D	0.933
LFD vs WD	0.902
n3D vs WD	0.723
LFD	
16 weeks vs. 1 year	0.078
n3D	
16 weeks vs. 1 year	0.059
WD	
16 weeks vs. 1 year	0.858

Panel E (Female gWAT TG uptake)

	# of animals
LFD	
16 weeks	8
1 year	7 (8-1 mouse died=7)
n3D	
16 weeks	8
1 year	6 (8-2 mice died=6)
WD	
16 weeks	8
1 year	7 (8-1 mouse died=7)
	P value
2-way ANOVA	
Age	0.006**
Diet	0.325
Tukey's multiple comparison	
16 weeks	
LFD vs n3D	0.139
LFD vs WD	0.961
n3D vs WD	0.081
1 year	
LFD vs n3D	0.796
LFD vs WD	0.750
n3D vs WD	0.999
LFD	
16 weeks vs. 1 year	0.009**
n3D	
16 weeks vs. 1 year	0.880
WD	
16 weeks vs. 1 year	0.031*

Panel F (Female BAT TG uptake)

	# of animals
LFD	
16 weeks	8
1 year	7 (8-1 mouse died=7)
n3D	
16 weeks	8
1 year	6 (8-2 mice died=6)
WD	
16 weeks	8

1 year	7 (8-1 mouse died=7)
	P value
2-way ANOVA	
Age	0.014*
Diet	0.102
Tukey’s multiple comparison	
16 weeks	
LFD vs n3D	0.860
LFD vs WD	0.347
n3D vs WD	0.653
1 year	
LFD vs n3D	0.408
LFD vs WD	0.705
n3D vs WD	0.112
LFD	
16 weeks vs. 1 year	0.488
n3D	
16 weeks vs. 1 year	0.017*
WD	
16 weeks vs. 1 year	0.225

Panel G (Male Heart TG uptake)

	# of animals
LFD	
16 weeks	8
1 year	8
n3D	
16 weeks	7 (8-1 outlier value=7)
1 year	7 (8-1 mouse died=7)
WD	
16 weeks	8
1 year	6 (8-2 outlier values=6)
	P value
2-way ANOVA	
Age	0.060
Diet	<0.001***
Tukey’s multiple comparison	
16 weeks	
LFD vs n3D	<0.001***
LFD vs WD	0.984
n3D vs WD	<0.001***
1 year	
LFD vs n3D	<0.001***
LFD vs WD	0.895
n3D vs WD	<0.001***
LFD	
16 weeks vs. 1 year	0.703
n3D	
16 weeks vs. 1 year	0.028*
WD	
16 weeks vs. 1 year	0.522

Panel H (Male Liver TG uptake)

	# of animals
LFD	
16 weeks	8
1 year	8
n3D	
16 weeks	8
1 year	6 (8-1 mouse died-1 outlier value=6)
WD	
16 weeks	8
1 year	8
	P value
2-way ANOVA	
Age	<0.001***
Diet	0.002**
Tukey's multiple comparison	
16 weeks	
LFD vs n3D	0.542
LFD vs WD	0.984
n3D vs WD	0.441
1 year	
LFD vs n3D	0.068
LFD vs WD	0.122
n3D vs WD	<0.001***
LFD	
16 weeks vs. 1 year	<0.001***
n3D	
16 weeks vs. 1 year	0.020*
WD	
16 weeks vs. 1 year	<0.001***

Panel I (Male Kidney TG uptake)

	# of animals
LFD	
16 weeks	8
1 year	8
n3D	
16 weeks	8
1 year	7 (8-1 mouse died=7)
WD	
16 weeks	8
1 year	8
	P value
2-way ANOVA	
Age	0.086
Diet	0.109
Tukey's multiple comparison	
16 weeks	
LFD vs n3D	0.982
LFD vs WD	0.608
n3D vs WD	0.495
1 year	
LFD vs n3D	0.671

LFD vs WD	0.563
n3D vs WD	0.166
LFD	
16 weeks vs. 1 year	0.419
n3D	
16 weeks vs. 1 year	0.150
WD	
16 weeks vs. 1 year	0.461

Panel J (Male Quadriceps TG uptake)

	# of animals
LFD	
16 weeks	8
1 year	8
n3D	
16 weeks	8
1 year	7 (8-1 mouse died=7)
WD	
16 weeks	8
1 year	8
	P value
2-way ANOVA	
Age	0.032*
Diet	<0.001***
Tukey's multiple comparison	
16 weeks	
LFD vs n3D	0.153
LFD vs WD	0.999
n3D vs WD	0.157
1 year	
LFD vs n3D	0.025*
LFD vs WD	0.257
n3D vs WD	<0.001***
LFD	
16 weeks vs. 1 year	0.291
n3D	
16 weeks vs. 1 year	0.893
WD	
16 weeks vs. 1 year	0.010*

Panel K (Male gWAT TG uptake)

	# of animals
LFD	
16 weeks	8
1 year	8
n3D	
16 weeks	8
1 year	7 (8-1 mouse died=7)
WD	
16 weeks	8
1 year	8
	P value
2-way ANOVA	

Age	<0.001***
Diet	0.455
Tukey's multiple comparison	
16 weeks	
LFD vs n3D	0.527
LFD vs WD	0.431
n3D vs WD	0.062
1 year	
LFD vs n3D	0.778
LFD vs WD	0.180
n3D vs WD	0.539
LFD	
16 weeks vs. 1 year	<0.001***
n3D	
16 weeks vs. 1 year	<0.001***
WD	
16 weeks vs. 1 year	0.268

Panel L (Male BAT TG uptake)

	# of animals
LFD	
16 weeks	8
1 year	8
n3D	
16 weeks	8
1 year	7 (8-1 mouse died=7)
WD	
16 weeks	8
1 year	8
	P value
2-way ANOVA	
Age	0.057
Diet	<0.001***
Tukey's multiple comparison	
16 weeks	
LFD vs n3D	0.561
LFD vs WD	0.302
n3D vs WD	0.040*
1 year	
LFD vs n3D	0.001**
LFD vs WD	0.603
n3D vs WD	<0.001***
LFD	
16 weeks vs. 1 year	0.986
n3D	
16 weeks vs. 1 year	0.007**
WD	
16 weeks vs. 1 year	0.608

Supplementary Figure 7 statistics

Panel A (Female Heart TG uptake)

	# of animals
LFD	
16 weeks	8
1 year	6 (8-1 mouse died-one outlier value=6)
2 years	6 (10-4 mice died=6)
n3D	
16 weeks	7 (8-1 outlier value=7)
1 year	6 (8-2 mice died=6)
2 years	3 (10-7 mice died=3)
WD	
16 weeks	8
1 year	7 (8-1 mouse died=7)
2 years	0 (10-10 mice died=0)
	P value
2-way ANOVA	
Age	<0.001***
Diet	<0.001***
Tukey's multiple comparison	
16 weeks	
LFD vs n3D	<0.001***
LFD vs WD	0.512
n3D vs WD	<0.001***
1 year	
LFD vs n3D	0.043*
LFD vs WD	0.820
n3D vs WD	0.007**
2 years	
LFD vs n3D	0.009**
LFD	
16 weeks vs 1 year	0.959
16 weeks vs 2 years	0.983
1 year vs 2 years	0.995
n3D	
16 weeks vs 1 year	<0.001***
16 weeks vs 2 years	0.006**
1 year vs 2 years	0.604
WD	
16 weeks vs. 1 year	0.332

Panel B (Female Liver TG uptake)

	# of animals
LFD	
16 weeks	8
1 year	7 (8-1 mouse died=7)
2 years	6 (10-4 mice died=6)
n3D	
16 weeks	8
1 year	6 (8-2 mice died=6)
2 years	3 (10-7 mice died=3)
WD	

16 weeks	8
1 year	7 (8-1 mouse died=7)
2 years	0 (10-10 mice died=0)
	P value
2-way ANOVA	
Age	0.001**
Diet	<0.001***
Tukey's multiple comparison	
16 weeks	
LFD vs n3D	<0.001***
LFD vs WD	<0.001***
n3D vs WD	0.952
1 year	
LFD vs n3D	0.045*
LFD vs WD	0.028*
n3D vs WD	0.996
2 years	
LFD vs n3D	0.003**
LFD	
16 weeks vs 1 year	0.008**
16 weeks vs 2 years	0.093
1 year vs 2 years	0.674
n3D	
16 weeks vs 1 year	0.195
16 weeks vs 2 years	0.067
1 year vs 2 years	0.674
WD	
16 weeks vs. 1 year	0.238

Panel C (Female Kidney TG uptake)

	# of animals
LFD	
16 weeks	8
1 year	7 (8-1 mouse died=7)
2 years	6 (10-4 mice died=6)
n3D	
16 weeks	8
1 year	6 (8-2 mice died=6)
2 years	3 (10-7 mice died=3)
WD	
16 weeks	8
1 year	7 (8-1 mouse died=7)
2 years	0 (10-10 mice died=0)
	P value
2-way ANOVA	
Age	<0.001***
Diet	0.480
Tukey's multiple comparison	
16 weeks	
LFD vs n3D	0.136
LFD vs WD	0.903
n3D vs WD	0.290
1 year	

LFD vs n3D	0.145
LFD vs WD	0.032*
n3D vs WD	0.828
2 years	
LFD vs n3D	0.262
LFD	
16 weeks vs 1 year	0.992
16 weeks vs 2 years	0.574
1 year vs 2 years	0.522
n3D	
16 weeks vs 1 year	<0.001***
16 weeks vs 2 years	0.067
1 year vs 2 years	0.713
WD	
16 weeks vs. 1 year	0.006**

Panel D (Female Quadriceps TG uptake)

	# of animals
LFD	
16 weeks	8
1 year	7 (8-1 mouse died=7)
2 years	6 (10-4 mice died=6)
n3D	
16 weeks	7 (8-1 outlier value=7)
1 year	6 (8-2 mice died=6)
2 years	3 (10-7 mice died=3)
WD	
16 weeks	8
1 year	7 (8-1 mouse died=7)
2 years	0 (10-10 mice died=0)
	P value
2-way ANOVA	
Age	<0.001***
Diet	0.738
Tukey’s multiple comparison	
16 weeks	
LFD vs n3D	0.302
LFD vs WD	0.357
n3D vs WD	0.986
1 year	
LFD vs n3D	0.995
LFD vs WD	0.795
n3D vs WD	0.859
2 years	
LFD vs n3D	0.297
LFD	
16 weeks vs 1 year	0.302
16 weeks vs 2 years	<0.001***
1 year vs 2 years	0.003**
n3D	
16 weeks vs 1 year	0.995
16 weeks vs 2 years	0.402
1 year vs 2 years	0.379

WD	
16 weeks vs. 1 year	0.867

Panel E (Female gWAT TG uptake)

	# of animals
LFD	
16 weeks	8
1 year	7 (8-1 mouse died=7)
2 years	6 (10-4 mice died=6)
n3D	
16 weeks	8
1 year	6 (8-2 mice died=6)
2 years	3 (10-7 mice died=3)
WD	
16 weeks	8
1 year	7 (8-1 mouse died=7)
2 years	0 (10-10 mice died=0)
	P value
2-way ANOVA	
Age	0.007**
Diet	0.379
Tukey's multiple comparison	
16 weeks	
LFD vs n3D	0.333
LFD vs WD	0.473
n3D vs WD	0.032*
1 year	
LFD vs n3D	0.102
LFD vs WD	0.059
n3D vs WD	0.985
2 years	
LFD vs n3D	0.958
LFD	
16 weeks vs 1 year	0.949
16 weeks vs 2 years	0.624
1 year vs 2 years	0.812
n3D	
16 weeks vs 1 year	0.001**
16 weeks vs 2 years	0.297
1 year vs 2 years	0.328
WD	
16 weeks vs. 1 year	0.255

Panel F (Female sWAT TG uptake)

	# of animals
LFD	
16 weeks	8
1 year	7 (8-1 mouse died=7)
2 years	6 (10-4 mice died=6)
n3D	
16 weeks	8
1 year	6 (8-2 mice died=6)
2 years	3 (10-7 mice died=3)

WD	
16 weeks	8
1 year	7 (8-1 mouse died=7)
2 years	0 (10-10 mice died=0)
	P value
2-way ANOVA	
Age	0.002**
Diet	0.116
Tukey's multiple comparison	
16 weeks	
LFD vs n3D	0.946
LFD vs WD	0.483
n3D vs WD	0.311
1 year	
LFD vs n3D	0.063
LFD vs WD	0.040*
n3D vs WD	0.995
2 years	
LFD vs n3D	0.989
LFD	
16 weeks vs 1 year	0.884
16 weeks vs 2 years	0.902
1 year vs 2 years	0.668
n3D	
16 weeks vs 1 year	0.008**
16 weeks vs 2 years	0.963
1 year vs 2 years	0.029*
WD	
16 weeks vs. 1 year	0.137

Panel G (Female BAT TG uptake)

	# of animals
LFD	
16 weeks	8
1 year	7 (8-1 mouse died=7)
2 years	6 (10-4 mice died=6)
n3D	
16 weeks	8
1 year	6 (8-2 mice died=6)
2 years	3 (10-7 mice died=3)
WD	
16 weeks	8
1 year	7 (8-1 mouse died=7)
2 years	0 (10-10 mice died=0)
	P value
2-way ANOVA	
Age	<0.001***
Diet	0.183
Tukey's multiple comparison	
16 weeks	
LFD vs n3D	0.507
LFD vs WD	0.779
n3D vs WD	0.897

1 year	
LFD vs n3D	0.950
LFD vs WD	0.069
n3D vs WD	0.154
2 years	
LFD vs n3D	0.292
LFD	
16 weeks vs 1 year	0.863
16 weeks vs 2 years	0.382
1 year vs 2 years	0.187
n3D	
16 weeks vs 1 year	0.972
16 weeks vs 2 years	0.003**
1 year vs 2 years	0.007**
WD	
16 weeks vs. 1 year	0.078

<u>Supplementary Figure 8 statistics</u>

Panel A (Male Heart TG uptake)

	# of animals
LFD	
16 weeks	7 (8-1 outlier value=7)
1 year	8
2 years	5 (8-3 mice died=5)
n3D	
16 weeks	8
1 year	7 (8-1 mouse died=7)
2 years	5 (8-3 mice died=5)
WD	
16 weeks	8
1 year	7 (8-1 mouse died=7)
2 years	3 (8-5 mice died=3)
	P value
2-way ANOVA	
Age	0.001**
Diet	<0.001***
Tukey's multiple comparison	
16 weeks	
LFD vs n3D	<0.001***
LFD vs WD	0.826
n3D vs WD	<0.001***
1 year	
LFD vs n3D	<0.001***
LFD vs WD	0.979
n3D vs WD	<0.001***
2 years	
LFD vs n3D	0.015
LFD vs WD	0.434
n3D vs WD	0.422
LFD	
16 weeks vs 1 year	0.943
16 weeks vs 2 years	0.113

1 year vs 2 years	0.180
n3D	
16 weeks vs 1 year	0.994
16 weeks vs 2 years	<0.001***
1 year vs 2 years	<0.001***
WD	
16 weeks vs. 1 year	0.509
16 weeks vs 2 years	0.657
1 year vs 2 years	0.998

Panel B (Male Liver TG uptake)

	# of animals
LFD	
16 weeks	8
1 year	8
2 years	5 (8-3 mice died=5)
n3D	
16 weeks	8
1 year	6 (8-1 mouse died-1 outlier value=6)
2 years	5 (8-3 mice died=5)
WD	
16 weeks	8
1 year	7 (8-1 mouse died=7)
2 years	3 (8-5 mice died=3)
	P value
2-way ANOVA	
Age	<0.001***
Diet	<0.001***
Tukey's multiple comparison	
16 weeks	
LFD vs n3D	0.008**
LFD vs WD	0.039*
n3D vs WD	0.824
1 year	
LFD vs n3D	0.007**
LFD vs WD	<0.001***
n3D vs WD	0.096
2 years	
LFD vs n3D	0.289
LFD vs WD	0.557
n3D vs WD	0.956
LFD	
16 weeks vs 1 year	0.973
16 weeks vs 2 years	<0.001***
1 year vs 2 years	<0.001***
n3D	
16 weeks vs 1 year	0.874
16 weeks vs 2 years	0.003**
1 year vs 2 years	0.016*
WD	
16 weeks vs. 1 year	0.004**
16 weeks vs 2 years	0.009**
1 year vs 2 years	0.860

Panel C (Male Kidney TG uptake)

	# of animals
LFD	
16 weeks	8
1 year	8
2 years	5 (8-3 mice died=5)
n3D	
16 weeks	8
1 year	7 (8-1 mouse died=7)
2 years	5 (8-3 mice died=5)
WD	
16 weeks	8
1 year	7 (8-1 mouse died=7)
2 years	3 (8-5 mice died=3)
	P value
2-way ANOVA	
Age	0.052
Diet	0.002**
Tukey's multiple comparison	
16 weeks	
LFD vs n3D	0.434
LFD vs WD	0.374
n3D vs WD	0.993
1 year	
LFD vs n3D	0.683
LFD vs WD	0.103
n3D vs WD	0.453
2 years	
LFD vs n3D	0.273
LFD vs WD	0.023*
n3D vs WD	0.354
LFD	
16 weeks vs 1 year	0.403
16 weeks vs 2 years	0.762
1 year vs 2 years	0.166
n3D	
16 weeks vs 1 year	0.650
16 weeks vs 2 years	0.998
1 year vs 2 years	0.674
WD	
16 weeks vs. 1 year	0.115
16 weeks vs 2 years	0.369
1 year vs 2 years	0.981

Panel D (Male Quadriceps TG uptake)

	# of animals
LFD	
16 weeks	8
1 year	8
2 years	5 (8-3 mice died=5)
n3D	
16 weeks	8
1 year	7 (8-1 mouse died=7)

2 years	5 (8-3 mice died=5)
WD	
16 weeks	8
1 year	7 (8-1 mouse died=7)
2 years	3 (8-5 mice died=3)
	P value
2-way ANOVA	
Age	<0.001***
Diet	0.003**
Tukey's multiple comparison	
16 weeks	
LFD vs n3D	0.028*
LFD vs WD	0.895
n3D vs WD	0.079
1 year	
LFD vs n3D	0.056
LFD vs WD	0.909
n3D vs WD	0.025*
2 years	
LFD vs n3D	0.954
LFD vs WD	0.570
n3D vs WD	0.419
LFD	
16 weeks vs 1 year	0.056
16 weeks vs 2 years	0.714
1 year vs 2 years	0.017*
n3D	
16 weeks vs 1 year	0.043*
16 weeks vs 2 years	0.446
1 year vs 2 years	0.577
WD	
16 weeks vs. 1 year	0.008**
16 weeks vs 2 years	0.725
1 year vs 2 years	0.256

Panel E (Male gWAT TG uptake)

	# of animals
LFD	
16 weeks	8
1 year	8
2 years	5 (8-3 mice died=5)
n3D	
16 weeks	8
1 year	7 (8-1 mouse died=7)
2 years	5 (8-3 mice died=5)
WD	
16 weeks	8
1 year	7 (8-1 mouse died=7)
2 years	3 (8-5 mice died=3)
	P value
2-way ANOVA	
Age	<0.001***
Diet	<0.001***

Tukey's multiple comparison	
16 weeks	
LFD vs n3D	0.550
LFD vs WD	0.006**
n3D vs WD	0.087
1 year	
LFD vs n3D	0.171
LFD vs WD	0.035
n3D vs WD	0.752
2 years	
LFD vs n3D	0.489
LFD vs WD	0.011
n3D vs WD	<0.001***
LFD	
16 weeks vs 1 year	0.054
16 weeks vs 2 years	0.802
1 year vs 2 years	0.024*
n3D	
16 weeks vs 1 year	0.009**
16 weeks vs 2 years	0.018*
1 year vs 2 years	<0.001***
WD	
16 weeks vs. 1 year	0.193
16 weeks vs 2 years	0.938
1 year vs 2 years	0.589

Panel F (Male sWAT TG uptake)

	# of animals
LFD	
16 weeks	8
1 year	8
2 years	5 (8-3 mice died=5)
n3D	
16 weeks	8
1 year	7 (8-1 mouse died=7)
2 years	5 (8-3 mice died=5)
WD	
16 weeks	8
1 year	7 (8-1 mouse died=7)
2 years	3 (8-5 mice died=3)
	P value
2-way ANOVA	
Age	<0.001***
Diet	0.008**
Tukey's multiple comparison	
16 weeks	
LFD vs n3D	0.615
LFD vs WD	0.585
n3D vs WD	0.139
1 year	
LFD vs n3D	0.461
LFD vs WD	0.297
n3D vs WD	0.952

2 years	
LFD vs n3D	0.030*
LFD vs WD	0.020*
n3D vs WD	0.862
LFD	
16 weeks vs 1 year	0.550
16 weeks vs 2 years	0.011*
1 year vs 2 years	<0.001***
n3D	
16 weeks vs 1 year	0.008**
16 weeks vs 2 years	0.754
1 year vs 2 years	0.109
WD	
16 weeks vs. 1 year	0.272
16 weeks vs 2 years	0.962
1 year vs 2 years	0.334

Panel G (Male BAT TG uptake)

	# of animals
LFD	
16 weeks	8
1 year	8
2 years	5 (8-3 mice died=5)
n3D	
16 weeks	8
1 year	7 (8-1 mouse died=7)
2 years	5 (8-3 mice died=5)
WD	
16 weeks	8
1 year	7 (8-1 mouse died=7)
2 years	3 (8-5 mice died=3)
	P value
2-way ANOVA	
Age	<0.001***
Diet	0.460
Tukey's multiple comparison	
16 weeks	
LFD vs n3D	0.094
LFD vs WD	0.168
n3D vs WD	<0.001***
1 year	
LFD vs n3D	0.714
LFD vs WD	0.366
n3D vs WD	0.104
2 years	
LFD vs n3D	0.512
LFD vs WD	0.284
n3D vs WD	0.041*
LFD	
16 weeks vs 1 year	0.096
16 weeks vs 2 years	0.027*
1 year vs 2 years	<0.001***
n3D	

16 weeks vs 1 year	0.005**
16 weeks vs 2 years	0.901
1 year vs 2 years	0.040*
WD	
16 weeks vs. 1 year	0.239
16 weeks vs 2 years	<0.001***
1 year vs 2 years	<0.001***

<u>Supplementary Figure 9 statistics</u>

Panel A (Female GTT 2 years)

	# of animals
LFD	6 (10-4 mice died=6)
n3D	4 (10-6 mice died=4)
WD	2 (10-8 mice died=2)

Panel B (Male GTT 2 years)

	# of animals
LFD	6 (8-2 mice died=6)
n3D	5 (8-3 mice died=5)
WD	3 (8-5 mice died=3)

Panel C (female insulin 2 years)

	# of animals
LFD	6 (10-4 mice died=6)
n3D	4 (10-6 mice died=4)
WD	2 (10-8 mice died=2)

<u>Supplementary Figure 10 statistics</u>

Panel A (Female ITT 2 years)

	# of animals
LFD	6 (10-4 mice died=6)
n3D	4 (10-6 mice died=4)
WD	2 (10-8 mice died=2)

Panel B (Male ITT 2 years)

	# of animals
LFD	6 (8-2 mice died=6)
n3D	5 (8-3 mice died=5)
WD	3 (8-5 mice died=3)
